# Unraveling the Relevance of ARL GTPases in Cutaneous Melanoma Prognosis through Integrated Bioinformatics Analysis

**DOI:** 10.3390/ijms22179260

**Published:** 2021-08-26

**Authors:** Cheila Brito, Bruno Costa-Silva, Duarte C. Barral, Marta Pojo

**Affiliations:** 1 Unidade de Investigação em Patobiologia Molecular (UIPM) do Instituto Português de Oncologia de Lisboa Francisco Gentil E.P.E., Rua Prof. Lima Basto, 1099-023 Lisbon, Portugal; cheila__brito@hotmail.com; 2 Champalimaud Research, Champalimaud Centre for the Unknown, Avenida de Brasília, 1400-038 Lisbon, Portugal; bruno.costadasilva@research.fchampalimaud.org; 3 iNOVA4Health, CEDOC, NOVA Medical School, NMS, Universidade NOVA de Lisboa, 1169-056 Lisbon, Portugal; duarte.barral@nms.unl.pt

**Keywords:** ADP-ribosylation factor-like (ARL), small GTPases, cutaneous melanoma, biomarkers, prognosis, immune microenvironment, metastasis, in silico study

## Abstract

Cutaneous melanoma (CM) is the deadliest skin cancer, whose molecular pathways underlying its malignancy remain unclear. Therefore, new information to guide evidence-based clinical decisions is required. Adenosine diphosphate (ADP)-ribosylation factor-like (ARL) proteins are membrane trafficking regulators whose biological relevance in CM is undetermined. Here, we investigated *ARL* expression and its impact on CM prognosis and immune microenvironment through integrated bioinformatics analysis. Our study found that all 22 *ARLs* are differentially expressed in CM. Specifically, *ARL1* and *ARL11* are upregulated and *ARL15* is downregulated regardless of mutational frequency or copy number variations. According to TCGA data, *ARL1* and *ARL15* represent independent prognostic factors in CM as well as *ARL11* based on GEPIA and OncoLnc. To investigate the mechanisms by which *ARL1* and *ARL11* increase patient survival while *ARL15* reduces it, we evaluated their correlation with the immune microenvironment. CD4^+^ T cells and neutrophil infiltrates are significantly increased by *ARL1* expression. Furthermore, *ARL11* expression was correlated with 17 out of 21 immune infiltrates, including CD8^+^ T cells and M2 macrophages, described as having anti-tumoral activity. Likewise, ARL11 is interconnected with ZAP70, ADAM17, and P2RX7, which are implicated in immune cell activation. Collectively, this study provides the first evidence that *ARL1*, *ARL11*, and *ARL15* may influence CM progression, prognosis, and immune microenvironment remodeling.

## 1. Introduction

Cutaneous melanoma (CM) is the most invasive type of skin cancer, accounting for 60% to 75% of the mortality rate related to skin neoplasms [1,2]. The dismal prognosis of this pathology is mainly associated with its elevated metastatic potential [3]. CM often shows the ability to metastasize even when primary lesions are thinner than 1 mm, presenting high aggressiveness and poor prognosis [3,4]. According to the melanoma research alliance statistics, stage IV CM is characterized by a 5-year survival rate of only 22.5% and there is evidence showing that patients with three or more sites of metastatic disease die within 1 year [5,6]. Therefore, efforts have been made toward the early diagnosis of CM, as early-stage CM presents a 5-year survival rate of around 90% [7]. 

Only in the past few years, the overall survival of CM patients has markedly improved with the introduction of targeted therapies and immunotherapies [8,9,10,11]. Unfortunately, treatment failures, adverse side effects, and acquired resistances represent the main causes of the limited success of these therapeutic approaches [12,13,14]. Hence, the metastatic ability and mechanisms of resistance to therapy render the treatment of this disease challenging. 

Presently, the molecular mechanisms and strategies of immune evasion essential for CM spreading are not well understood [15]. Early identification of aggressive CM remains one of the main goals of CM research. In this regard, the identification of new biomarkers of prognosis and metastasis is still awaited to allow patient stratification based on disease malignancy. Accordingly, the knowledge about the dysregulated signaling pathways contributing to CM progression may provide important clues for the development of novel and efficient therapies [16]. 

The adenosine diphosphate (ADP)-ribosylation factor (Arf) family of proteins belongs to the Ras superfamily of small GTPases, whose members are responsible for the regulation of essential physiological functions such as cell signaling, membrane trafficking, and cytoskeleton reorganization [17,18]. The Arf family comprises around 30 members, namely 5 ARFs and 22 ARF-like (ARLs) proteins in humans [19]. These low-molecular-weight proteins alternate between an inactive GDP-bound and an active GTP-bound state, working as molecular switches that regulate trafficking and signaling networks. Several studies have reported that ARFs can be subverted in various cancer types, influencing their malignancy [20,21,22]. For instance, ARF1, ARF3, and ARF4 are upregulated in breast cancer, promoting cell proliferation and migration [20,21,22,23]. Among ARF proteins, only ARF6 was described as having an essential role in early and late stages of CM metastasis, mediated by the activation of mitogen-activated protein kinase (MAPK) and phosphatidylinositol 3-kinase (PI3K)/protein kinase B(Akt) signaling pathways [24,25]. 

In contrast to Arf proteins, the biochemical and functional characterization of Arls under physiological and pathological conditions is poorly explored [26]. The physiological functions of most Arls are still unclear. Available data demonstrate that these proteins are involved in a multiplicity of functions. For instance, Arl1 is recruited to the trans-Golgi network being involved in multiple processes such as cargo transport, cell polarity, innate immunity, and secretion of insulin and matrix metalloproteinases [27]. Arl13b interacts with actin and it is also a regulator of ciliogenesis [28]. Furthermore, a recent study proposed that ARL11 is required for macrophage activation and immune function [29], and ARL15 has been described as having an important role in adipocyte differentiation and adiponectin secretion [30]. It is also known that *ARL2* is a tumor suppressor gene in breast and pancreatic cancer, but in cervical and bladder cancer its suppression is related to a significant decrease in cell proliferation, migration, and invasion [31,32,33]. *ARL4C* overexpression was reported as a prognostic factor of poor outcome in colorectal cancer and suggested as an important stimulator of cell proliferation and migration in several types of cancer [34,35]. Furthermore, *ARL8B* knockdown abrogates the growth of prostate tumors in mice [36], as well as decreases invasive tumor growth and distant metastasis of breast tumors in a mouse xenograft model [37] and *ARL5A* downregulation reduces colorectal cancer proliferation [38]. ARL13B, one of the most studied proteins from this family, was implicated in tumorigenesis and progression of medulloblastoma [39] and gastric [40] and breast [41] cancers. Indeed, our group has been focused on the study of ARLs for a long time, namely on the effect of ARL13B on breast cancer cell migration and invasion through cytoskeleton-related mechanisms [41]. In familial breast cancer and CM, *ARL11* was suggested as a low-penetrance tumor suppressor gene [42,43]. To the best our knowledge, this is the only study exploring the role of an *ARL* gene in CM. Considering the multiplicity of physiological processes in which ARL proteins are involved, and their impact on the growth and metastatic ability of several types of cancer, it is likely that they play important roles in CM tumorigenesis and progression [26]. However, the role of these proteins in cancer remains poorly explored, and specifically in CM, there are almost no data about the relevance of ARL GTPases. Thus, it is essential to understand their usefulness as potential biomarkers.

As far as we know, this is the first study based on integrated bioinformatics analysis to investigate the expression profile of *ARL* genes in primary and metastatic CM, their potential prognostic value, and biological roles in CM. Moreover, to identify the mechanisms by which *ARL* expression affects the survival rates of CM patients, we also assessed their influence on the immune microenvironment. Protein–protein interaction (PPI) networks were generated to identify important players whose interaction with ARLs could potentially affect CM prognosis. The bioinformatics analysis performed shows that *ARLs* are differentially expressed in primary and metastatic CM, which could be related to regulatory epigenetic mechanisms such as promoter methylation levels. Additionally, the survival analyses performed demonstrate that *ARL1*, *ARL11*, and *ARL15* expression represent independent prognostic factors in CM. Interestingly, ARL11 seems to be the prognostic factor with the most predominant impact on immune microenvironment remodeling and on the recruitment and activation of immune cells. Therefore, our systematic analysis provides an integrated understanding of ARL1, ARL11, and ARL15 function in CM and their usefulness as potential biomarkers for the prognosis of CM patients.

## 2. Results

### 2.1. ARL Genes Are Differentially Expressed in Primary and Metastatic CM

**Figure 1:**
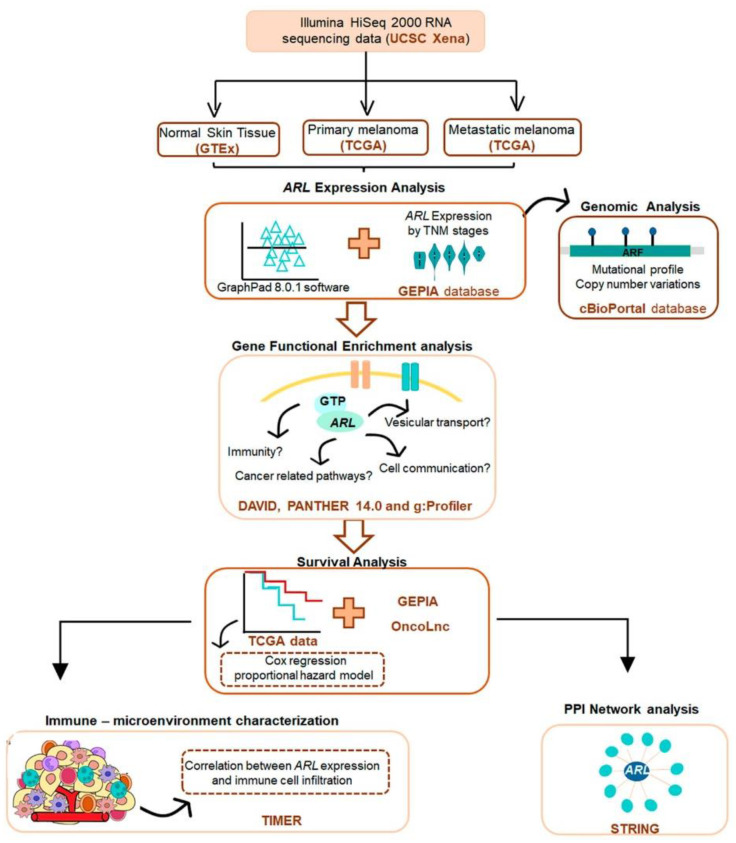
Study design and workflow of the integrated bioinformatics analysis used to evaluate ARL function in CM. Expression data were downloaded from the UCSC Xena project, which provides RNA sequencing data obtained from the TCGA and GTEx databases. *ARL* expression analyses were performed using TCGA and GTEx data, as well as Gene Expression Profiling Interactive Analysis (GEPIA) web server. Genomic analyses of *ARL* genes were implemented using cBioPortal. Gene functional enrichment analyses were accomplished using the Database for Annotation, Visualization and Integrated Discovery (DAVID), PANTHER 14.0, and g:Profiler software. Clinical data available on the TCGA database, OncoLnc, and GEPIA were used to perform the survival analyses included in this study. Immune-microenvironment relationship with *ARL* expression was estimated using Tumor Immune Estimation Resource (TIMER 2.0), and the Search Tool for the Retrieval of Interacting Genes (STRING) was also applied to discover ARL protein interaction networks.

Considering the lack of data on ARL function in CM, we evaluated their transcriptional levels in primary and metastatic CM samples using the Cancer Genome Atlas (TCGA) and Genotype tissue expression (GTEx) data, obtained from the University of California, Santa Cruz (UCSC) Xena project and processed using a uniform bioinformatic pipeline. The clinical characterization of patients with primary and metastatic CM is represented in Table S1. A detailed workflow of the study design is shown in Figure 1. 

**Figure 2:**
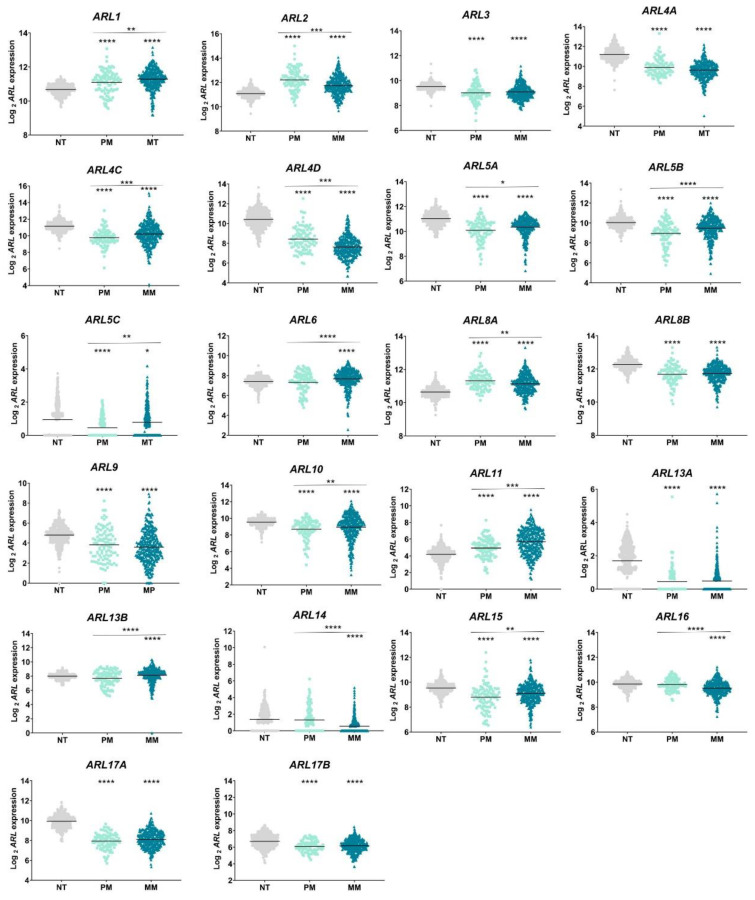
Transcriptional levels of the 22 *ARL* genes expressed in the human genome and identified in primary (PM) and metastatic (MM) melanoma samples, obtained from TCGA, were compared to their levels in normal skin tissue (NT) extracted from the GTEx database after being processed using a uniform bioinformatics pipeline. The Kruskal–Wallis tests with post hoc Dunn’s method were performed for multiple comparisons. All tests were two-sided, with a significance level of 5%. Graphical representations were performed using GraphPad Prism 8.4.3. * *p* < 0.05; ** *p* < 0.01; *** *p* < 0.001; **** *p* < 0.0001.

This analysis demonstrated that all 22 *ARLs* are differentially expressed in CM samples, as compared to normal skin tissue (Figure 2). *ARL1, ARL2, ARL8A*, and *ARL11* are significantly upregulated in primary and metastatic CM samples, while *ARL3, ARL4A, ARL4C, ARL4D, ARL5A, ARL5B, ARL5C, ARL8B, ARL9, ARL10, ARL13A, ARL13B*, *ARL15, ARL17A*, and *ARL17B* are downregulated (Figure 2). Interestingly, *ARL6* and *ARL13B* expression is significantly higher in metastatic CM, whereas *ARL14* and *ARL16* expression is decreased, suggesting their specific dysregulation in this stage of CM.

*ARL* expression was also evaluated by tumor staging (I–IV) using the GEPIA platform. This analysis validated the TCGA results, as no statistically significant differences were found in *ARL3, ARL8B, ARL9, ARL13A, ARL17A* or *ARL17B* expression between primary and metastatic CM (Figure S1). In addition, GEPIA analysis also showed that *ARL5A, ARL5B, ARL6, ARL8A, ARL10*, and *ARL14* expression is not correlated with SKCM pathological stages. The differences in these two subsets of analysis could be related to the statistical tests performed.

### 2.2. Promoter Methylation Levels May Influence the Expression of Some ARL Genes

Then, we verified whether the dysregulation of these 22 genes in CM could be related to their mutational rate or the presence of copy number variations, using cBioPortal. Surprisingly, these genes present a reduced mutational frequency ranging from 0% to 1.6% (Figure S2). *ARL11* (1.6%) and *ARL16* (1.6%) are the two most frequently mutated genes among the group evaluated (Figure S2). It is important to highlight that all the variants identified in these genes were reported as mutations in cBioPortal, although their pathogenicity was not determined yet.

Similarly, the presence of copy number variations in *ARL* genes is not common in CM samples (Figure S3). From the 367 samples assessed, 5% (19/367) contain amplifications in *ARL8A*, 3% (11/367) amplifications and deletions in *ARL2,* and 5% (18/367) present amplifications in *ARL16* (Figure S3). Additionally, promoter methylation analysis using the UALCAN tool demonstrated that *ARL3, ARL5A, ARL9, ARL13A, ARL15, ARL16, ARL17A,* and *ARL17B* present higher β-values in metastatic CM samples compared to their levels in normal tissue (Figure S4). These results suggest that the downregulation of these genes in metastatic CM samples could be related to epigenetic regulatory mechanisms. Despite the referred differences, the few genes presenting β-values ranging from 0.5 to 0.7, and thus considered hypermethylated, were *ARL4C, ARL5C, ARL9, ARL11,* and *ARL13A* (Figure S4). From these genes, all of them, except *ARL11*, were found downregulated in CM. Accordingly, these data-driven results suggest that the mutational rate and copy number variation status are not responsible for *ARL* dysregulation in CM. However, for some of these genes, epigenetic mechanisms, such as promoter methylation, may be implicated in the regulation of their expression in CM.

### 2.3. ARL GTPases Are Implicated in Cell Communication, Vesicle Transport, and Protein Recruitment in CM 

**Figure 3:**
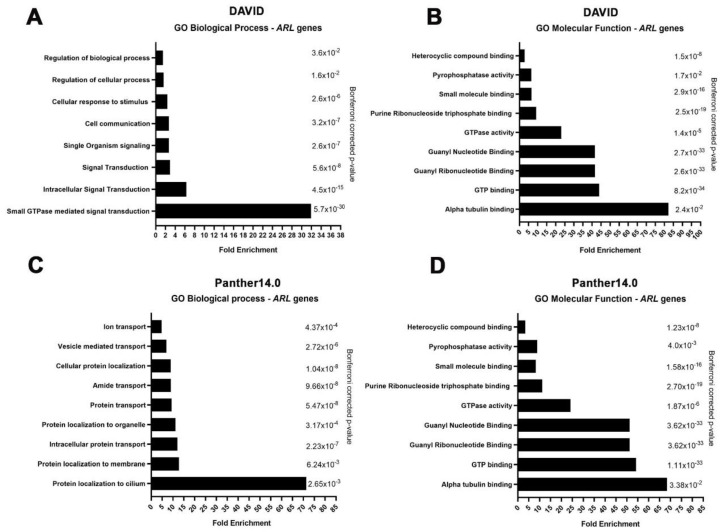
Gene ontology and biological pathway functional enrichment analyses of *ARL* genes using DAVID and PANTHER 14.0 against a *Homo sapiens* background reference. Biological processes (**A**) and molecular functions (**B**) enriched for *ARL* genes according to the DAVID web server. Biological processes (**C**) and molecular functions (**D**) enriched for *ARL* genes according to the PANTHER 14.0 web server. Statistical over-representation was calculated using a binomial test and the results were considered significant at *p* < 0.05 after Bonferroni correction.

Since we found that *ARLs* are differentially expressed in CM, we further investigated their involvement in relevant pathways related to cancer biology by employing gene ontology analyses. Importantly, *ARL* genes were found enriched in biological processes related to GTPase-mediated signaling transduction: cell communication; cellular response to stimuli; protein recruitment to the cilium; organelle, membrane, and vesicle-mediated transport; and intracellular protein transport (Figure 3A,C and Figure S5). Functionally, ARLs revealed an enrichment in processes related to α-tubulin binding, GTP binding, GTPase, and pyrophosphatase activity (Figure 3B,D).

### 2.4. ARL1, ARL11, and ARL15 Expression Represent Prognostic Factors in CM

**Figure 4:**
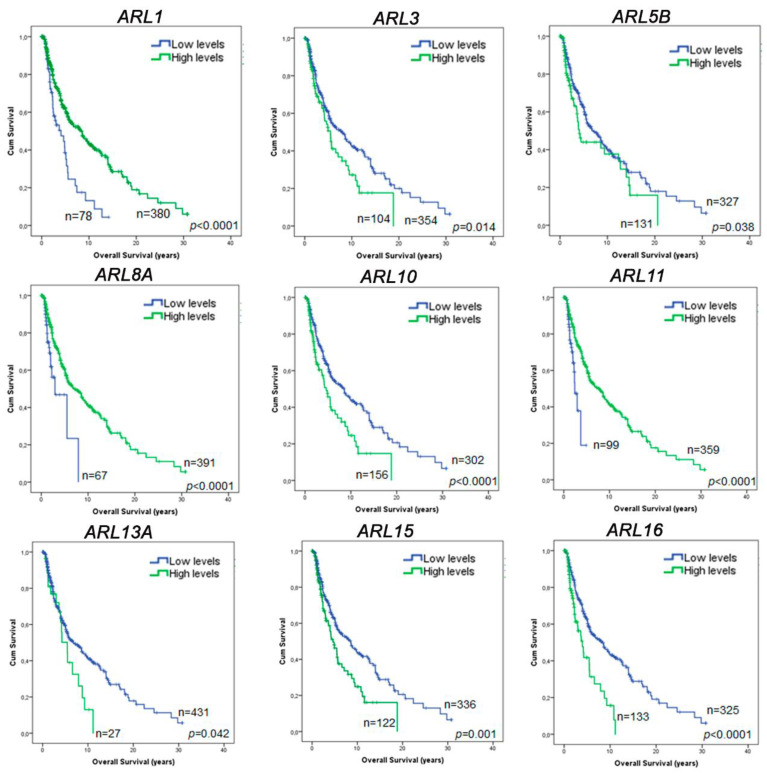
*ARL* prognostic value in CM patients assessed using TCGA clinical data. The results presented show exclusively the *ARL* genes whose expression is significantly correlated with CM patients’ overall survival. The Kaplan–Meier survival curves with higher (green) and lower (blue) *ARL* expression than the median expression found in normal skin tissue were generated using IBM SPSS Statistics 21.0. Differences in the overall survival of CM patients were identified by employing the log-rank test. *p* < 0.05 was considered statistically significant.

To unveil whether *ARLs* dysregulation can provide essential clinical insights, we explored their relevance in the prognosis of CM patients. Using clinical data of CM patients accessed from the TCGA database, we performed a survival analysis employing overall survival as the primary endpoint. From the 22 genes analyzed, only *ARL1, ARL3, ARL5B, ARL8A, ARL10, ARL11, ARL13A, ARL15,* and *ARL16* were found to have prognosis value in the univariable analysis (Figure 4 and Figure S6). Next, the Cox regression hazard model was performed using the following confounder variables: age, gender, tumor stage, type of tumor (primary/metastatic), and presence/absence of *BRAF, NRAS,* and *NF1* mutations. From the previous 9 genes with prognosis value in the univariable analysis, only 5 (*ARL1, ARL3, ARL10, ARL13A* and *ARL15*) correspond to independent prognostic factors for CM based on TCGA data (Tables S2–S10). An additional multivariable analysis performed using the expression of all *ARL* genes as confounding variables, in addition to the variables previously referred to, revealed that *ARL1* and *ARL15* are the only genes correlated with the overall survival of CM patients, emphasizing their relevance to CM prognosis (Table 1). The high *ARL1* expression group was found to exhibit a prolonged overall survival compared to the low *ARL1* expression group, while the low *ARL15* expression group is associated with a favorable prognosis.

Among all candidates, GEPIA and OncoLnc survival tools also show that a higher expression of *ARL11* is significantly associated with an improved prognosis of CM patients, agreeing with the survival analysis performed using TCGA data (Figures S7 and S8). Although *ARL11* is not an independent prognostic factor according to TCGA data, *ARL11* expression has independent prognosis value based on GEPIA and OncoLnc results. Hence, *ARL11* expression should also be considered a potential candidate with impact on CM prognosis. Altogether, these results highlight the role of *ARL1*, *ARL11,* and *ARL15* expression as important players in CM patient prognosis.

### 2.5. ARL11 May Have a Predominant Impact on Immune Microenvironment Remodeling

Since CM is a highly immunogenic type of tumor, after discovering that *ARL1*, *ARL11,* and *ARL15* expression may have a significant impact on CM patients’ prognosis, we investigated whether the immune microenvironment could be involved in the mechanisms associated with *ARL* prognostic value. For this purpose, the relationships between *ARL* expression and immune infiltrate levels in SKCM, SKCM-primary, and SKCM-metastasis datasets were determined. 

The integrative analysis of TIMER results showed that there is a significant general correlation between neutrophils, CD4^+^ T cells, and common lymphoid progenitor infiltration and *ARL1, ARL3, ARL4A, ARL4C, ARL5A, ARL5B, ARL11, ARL13B,* and *ARL15* expression. This suggests the existence of an immune signature characteristic of CM cells expressing these genes (Figures S9–S12). Therefore, when analyzing the significant correlations between all *ARL* genes and immune cell infiltrates, we discovered a common CM immune profile, which may be connected or even enhanced by *ARL* expression (Figures S9–S12). 

**Figure 5:**
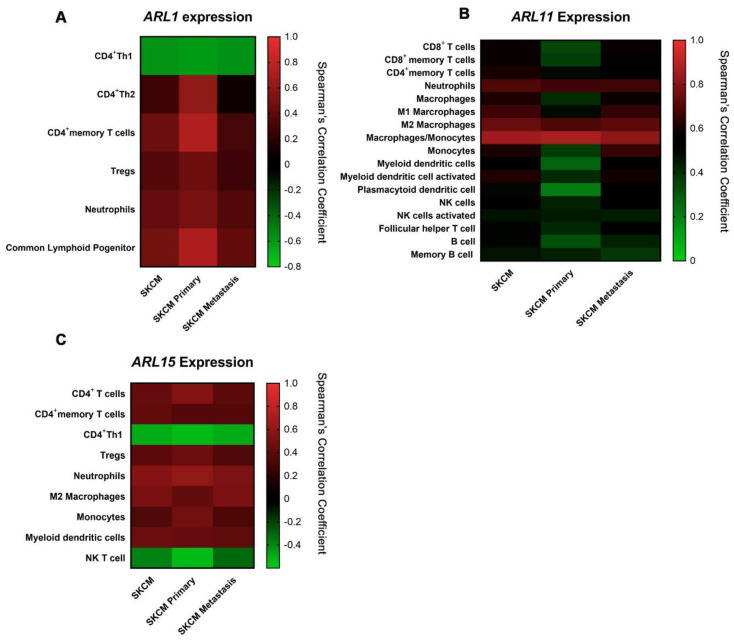
Spearman’s correlation coefficients calculated between immune cell infiltrates and *ARL1* (**A**), *ARL11* (**B**), and *ARL15* (**C**) expression in SKCM (*n* = 471), SKCM-primary, (*n* = 103) and SKCM-metastasis (*n* = 368) datasets. Data were obtained from the TIMER 2.0 platform and represented using heat maps. All the correlations indicated are statistically significant. Green is associated with weaker correlation coefficients, while red is indicative of stronger correlations. Correlations were determined using the purity adjustment definition and only genes with Spearman coefficient >0.4 were considered positively correlated.

Specifically, we observed that *ARL1* expression is negatively correlated with CD4^+^ Th1 cells (Cor = −0.601) and positively correlated with CD4^+^ Th2 cells (Cor = 0.598), neutrophils (Cor = 0.501), and common lymphoid progenitor cells (Cor = 0.720) in SKCM, being these correlations stronger in the SKCM-primary subset (Figure 5A and Figure S10). 

Interestingly, we found that *ARL11* expression is correlated with 17 out of the 21 immune infiltrates available on TIMER in either SKCM, SKCM-primary, or SKCM-metastasis datasets (Figure 5B and Figure S9). For instance, *ARL11* expression is positively correlated with CD8^+^ T cell infiltration (Cor = 0.576), using five distinct algorithms from the TIMER platform in SKCM samples, but particularly in the SKCM-metastasis subset (Table S11). Furthermore, specific positive correlations were identified with CD8^+^ memory T cells (Cor = 0.572), CD4^+^ memory T cells (Cor = 0.602), neutrophils (Cor = 0.697), macrophages (Cor = 0.603), M1 macrophages (Cor = 0.662), M2 macrophages (Cor = 0.739), monocytes (Cor = 0.842), myeloid dendritic cells (Cor = 0.624), myeloid dendritic cells activated (Cor = 0.612), plasmocytoid dendritic cells (Cor = 0.496), NK cells (Cor = 0.546), NK cells activated (Cor = 0.467), T cell follicular helper (Cor = 0.508), B cells (Cor = 0.498), and memory B cells (Cor = 0.463), using more than one algorithm for the majority of these immune subsets (Table S11). All the correlations highlighted are statistically significant. Based on these results, *ARL11* expression seems to be the prognostic factor with the most predominant function in immune microenvironment remodeling. Similarly to *ARL1*, *ARL15* expression is also negatively correlated with CD4^+^ Th1 cells (Cor = −0.506) and NK cells (Cor = −0.541) in the SKCM-primary dataset, while positively correlated with CD4^+^ T cells (Cor = 0.431), neutrophils (Cor = 0.559), and macrophages M2 (Cor = 0.504) in SKCM (Figure 5C and Figure S10).

### 2.6. ARL11 Is Closely Interconnected with Proteins Involved in Immune Cell Activation and Recruitment 

**Figure 6:**
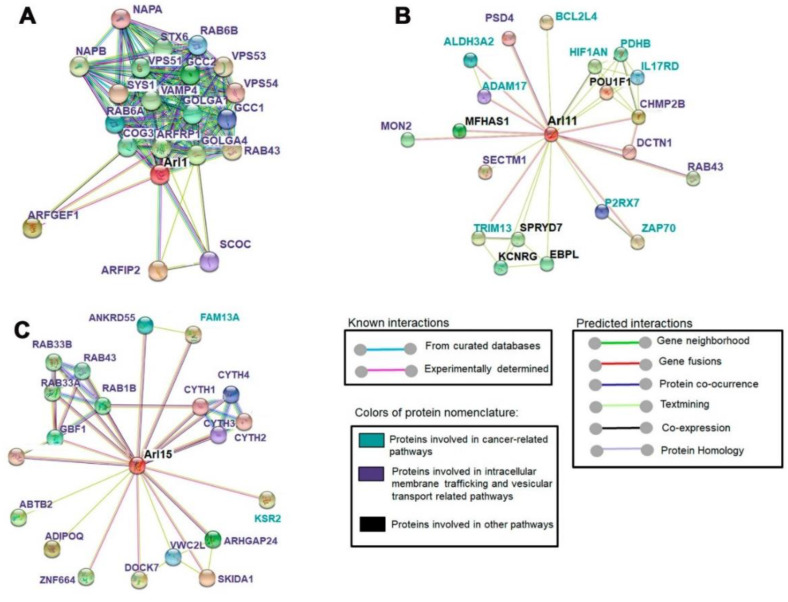
Protein–protein interaction networks containing the 20 proteins most related to ARL1 (**A**), ARL11 (**B**), and ARL15 (**C**) determined using STRING. Proteins in blue are involved in cancer-related pathways, while proteins in purple are involved in membrane trafficking and vesicular transport, and proteins in black are involved in other pathways. Scores between proteins were established based on their interaction confidence and existing evidence. Blue lines represent interactions predicted from curated databases, whereas pink lines refer to interactions experimentally determined.

Protein–protein interaction networks were generated through SPRING version 11 database, choosing the top 20 most related proteins to ARL1, ARL11, and ARL15. According to this analysis, ARL1 and ARL15 are mainly associated with proteins involved in membrane trafficking and vesicular transport-related pathways (Figure 6A,C). The scores of ARL1 interactions range between 0.921 and 0.998, while the ARL15-associated network achieves scores between 0.6 and 0.7 (Figure 6A,C). Additionally, we found that ARL11 is interconnected with a set of proteins such as ZAP70, interleukin-17 receptor D (IL17RD), disintegrin and metalloproteinase domain-containing protein 17 (ADAM17), Bcl-2-like protein 14 (BCL2L4), E3 ubiquitin-protein ligase (TRIM13), P2X purinoceptor 7 (P2Rx7), and hypoxia-inducible factor 1-alpha inhibitor (HIF1AN) (scores from 0.5 to 0.7), all implicated in cancer-related pathways (Figure 6B). Hence, these interactions highlight the involvement of ARL11 in mechanisms implicated in innate and adaptive immune activation, caspase activation, Ras-MAPK regulation, inflammation, and tumorigenesis. 

## 3. Discussion

Despite the remarkable efforts to improve our understanding of CM biology, there is still a need to identify new biomarkers to further stratify patients based on prognosis, metastatic propensity, and response to therapy [15]. Unfortunately, current knowledge about ARF family members in CM is limited, and in particular, ARL proteins’ involvement in CM etiology and progression remains unknown [26]. Hence, we evaluated the effect of *ARL* expression in prognosis, immune microenvironment modification, and their functional interactions with well-established signaling pathways in CM by performing an integrative analysis of open access databases. The large number of candidates included in this GTPase family led us, in a first stage, to perform a bioinformatics analysis to select the most clinically and biologically relevant Arls to be studied using in vitro and in vivo assays.

Analysis of transcriptomes obtained from TCGA and GTEx revealed that 22 *ARL* genes are differentially expressed in CM—4 upregulated and 14 downregulated in both primary and metastatic CM samples—while the remaining 4 genes are only found dysregulated in metastatic CM. Until now, no study has explored simultaneously the expression levels of these 22 genes in a cancer context. Similar to what was observed in CM, *ARL2* upregulation was also described in bladder [32] and cervical [31] cancers, as well as in hepatocellular carcinomas [44]. Furthermore, *ARL3* downregulation was verified in glioma [45]. While in CM *ARL11* was found upregulated, compared to normal skin tissue, in breast, lung, ovarian and prostate cancers, it was shown to be downregulated by DNA hypermethylation and genomic deletions [43,46]. The studies mentioned above investigated the function of single *ARL* genes. Hence, much remains to be known about the impact of the remaining *ARL* candidates on these types of cancer. Considering the expression profiles obtained, we hypothesized that *ARLs* may have essential functions during CM progression, probably representing promising biomarkers to distinguish malignant stages. 

To infer the main cause of *ARL* dysregulation in CM, we assessed the mutational rate and copy number alterations of these genes, although these events are not responsible for the differences identified in expression. Additionally, we evaluated promoter methylation levels to understand whether epigenetic mechanisms could be related to *ARL* differential expression. Despite the reduced number of skin samples (*n* = 2) included in the methylation analysis, it seems that this epigenetic event can be involved in the transcriptional regulation of these genes. Therefore, it is necessary to explore whether the *ARL* expression pattern is associated with the effect of driver genes or is even due to alterations in specific chromatin regulators, as previously described for other pathologies [47,48]. Further studies using well-characterized cohorts will be crucial to validate whether this expression profile is maintained at the protein level, providing relevant data for the clinical management of CM patients. 

Several studies have reported that dysregulation of *ARL* expression or activity can be associated with enhanced cell migration, invasion, and proliferation in distinct types of cancer [36,39,40,41,49]. Since gene ontology analyses demonstrated that ARLs are enriched for pathways implicated in the response to stimuli, protein and vesicle transport, signaling transduction, and cell communication, their involvement in CM cell migration and invasion seems possible. Recently, vesicle trafficking pathways have emerged as key regulatory elements in migration and invasion, with endocytosis and recycling of cell surface cargoes being of major importance [50,51]. Thus, the role of ARL proteins in CM malignant characteristics should be assessed using migration and invasion assays. 

Although some well-established biomarkers have no impact on CM prognosis, we decided to evaluate whether *ARL* genes could provide relevant knowledge about patients’ overall survival. Increased expression of *ARL1* and *ARL11* in CM is associated with a favorable prognosis, while low expression of *ARL15* is indicative of the poorest outcomes according to the clinical data available on the TCGA database. Consistently, a previous study proposed *ARL11* as a tumor suppressor gene due to its ability to inhibit tumor formation in immunodeficient mice after transfection with a lung cancer cell line [46]. Hence, the few available data on *ARL11* function are consistent with the survival results presented, evidencing the pivotal role of this gene in patients’ outcome. However, the functions of Arl11 and Arl15 under physiological conditions remain unknown.

Even though distinct factors influence CM occurrence and prognosis, immune responses are considered key factors owing to CM immunogenicity [52,53]. Additionally, the relationship between specific molecular signatures and immune microenvironment has been mentioned as contributing to tumor aggressiveness, consequently influencing patients’ prognosis and response to therapy [54]. Nevertheless, no study has examined the correlation between *ARL* expression and immune cell infiltration in CM. Hence, we sought to better understand this relationship and how it might affect CM prognosis. For instance, we verified that high *ARL1* expression enhances neutrophil, CD4^+^ Th2 cell and CLP infiltration, an immune profile that may be associated with an anti-tumoral effect, which is consistent with its physiological role in immune activation. Recent studies have shown the existence of a highly specific subset of neutrophils with an anti-tumoral activity related to the production of extracellular traps that inhibit CM cell migration [55]. In fact, neutrophils recruit and activate immune cells by producing a variety of chemical factors to stimulate T cell proliferation, NK, and dendritic cell maturation [54]. Additionally, a previous study also described that CD4^+^ Th2 cells can make the clearance of established lung and visceral CM metastases by enhancing CD8^+^ T cell activation, while CD4^+^ Th1 cells have no effect on tumor growth [56]. Another important immune subset includes CLP cells, which can differentiate into CD4^+^ Th cells, justifying why CLP and CD4^+^ Th2 cells are both correlated with *ARL1* expression [57]. Thus, the integrative analysis of these results leads us to hypothesize that *ARL1* upregulation positively affects the infiltration of these immune subsets, favoring an improved prognosis of CM patients.

**Figure 7:**
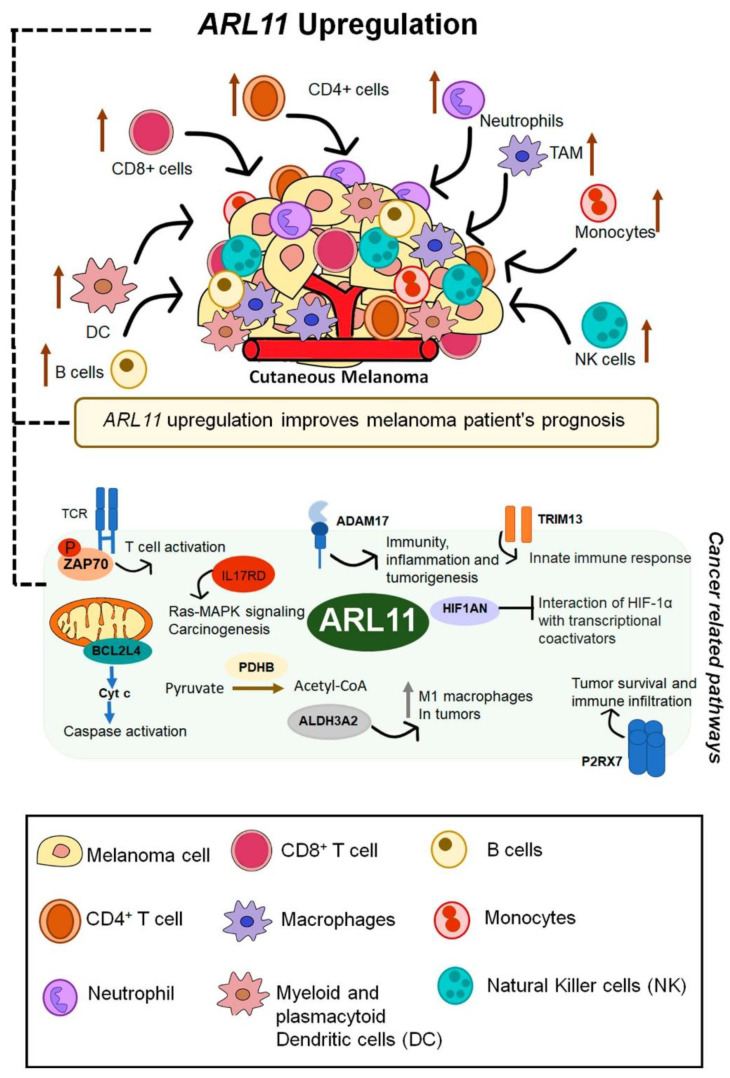
Schematic representation of *ARL11* involvement in immune microenvironment remodeling and its interactions with proteins involved in pathways of immune cell activation and recruitment, potentially associated with the mechanisms by which *ARL11* expression improves CM patients’ prognosis.

In addition to *ARL1*, *ARL11* is the most impactful gene contributing to CM immune microenvironment remodeling through the recruitment of CD8^+^ T cells, CD4^+^ memory T cells, macrophages, B cells, NK cells, neutrophils, and dendritic cells. Particularly, CD8^+^ T cells and M2 macrophages, which are characterized by their beneficial anti-tumoral effect, show very strong and reliable correlations with *ARL11* expression. Recently, a study found that peripheral CD8^+^ T cell characteristics are associated with more durable responses to immune checkpoint blockade in patients with metastatic CM [58]. There is also evidence showing that dendritic cells and some macrophages can prime adaptive immunity to incite cytotoxicity of CD8^+^ effector T cells [59]. In the last 5 years, many reports have recognized the critical role of CD4^+^ T cells in driving anti-tumor immunity and supporting anti-tumor CD8^+^ T cell responses [60]. Furthermore, evidence of tumor-resident mature B cells and reports of associations with favorable prognosis in malignant CM suggest that humoral immunity participates in anti-tumor defense [61]. Altogether, these results indicate that *ARL11* upregulation has a positive impact in CM patients’ overall survival, in part by inducing an immune profile relying on an anti-cancer activity to a greater extent compared to *ARL1* expression (Figure 7). However, given that there are few data about ARL function, tissue expression needs to be more thoroughly profiled because this is a major confounding factor in the analysis of immune cell infiltration in CM. Thus, the correlations between *ARL* expression and immune cell infiltrates observed using the TIMER web server should be further assessed using flow cytometry to ensure that these associations are specific of CM cells. This way, we can truly ensure that *ARL* expression in CM cells is driving increased infiltration of immune subsets and not that *ARL* are highly expressed in immune subsets rather than in tumor cells, which would yield the same observed associations.

The PPI analysis performed using the STRING database demonstrated the interaction between ARL11 and several proteins involved in innate and adaptative immune activation, corroborating the hypothesis that ARL11 is tightly interconnected with CM immune microenvironment modulation. For instance, ARL11 was associated with ZAP70 (score = 0.5), a cytoplasmic tyrosine kinase that plays a critical role in the events involved in initiating T cell responses by the antigen receptor [62]. Despite its controversial function, P2RX7, a plasma membrane receptor for extracellular ATP that is expressed at a high level by immune and tumor cells, also belongs to the PPI network of ARL11 (score = 0.7). New evidence suggests that P2RX7 has an essential role in restraining tumor progression [63]. Moreover, in vitro studies have shown that IL17RD, another ARL11-related protein (score = 0.7), exerts inhibitory effects on MAPK signaling to restrain the proliferation of various cancer cell lines [64]. Genes associated with MAPK regulation, caspase activation, and tumorigenesis are also part of the ARL11 interactive network, highlighting its pivotal role in the regulation of immune and cancer-related pathways (Figure 7). 

The integrated analysis of *ARL15* expression, prognosis, and immune characterization generated inconclusive data, because its correlation with immune cells is not sufficient to elucidate the putative mechanism related to *ARL15* prognosis value. However, the bioinformatics analysis performed highlighted the essential role of the tumor microenvironment in the mechanisms related to the prognostic value of *ARL1* expression but mostly *ARL11* expression. Further studies are required to validate whether the interplay between *ARL* expression and immune cell infiltration is truly reliable, ensuring the accuracy and clinical relevance of these assumptions. 

## 4. Conclusions

The understanding of CM biology is pivotal to gain insight about the mechanisms involved in its progression and simultaneously on how to impair these processes. Overall, this integrative in silico analysis suggests that *ARL* expression is highly dysregulated in CM, highlighting their relevance in CM tumorigenesis and metastasis. Additionally, this study underscores that *ARL11* expression may improve CM patient prognosis, mainly through the recruitment of immune infiltrates with anti-tumorigenic activity. Therefore, our systematic analysis provides an integrated understanding of the potential functions of ARL1, ARL11, and ARL15 in CM and their usefulness as biomarkers for the prognosis of CM patients. Further functional studies are needed to verify the validity of these findings.

## 5. Materials and Methods

### 5.1. Study Design and Data Acquisition

A detailed workflow of our study design is shown in Figure 1. Illumina HiSeq 2000 RNA sequencing data of CM and normal skin tissue samples from the Cancer Genome Atlas (TCGA) and Genotype tissue expression (GTEx), respectively, were downloaded from the University of California, Santa Cruz (UCSC) Xena project (http://xena.ucsc.edu) [65]. These datasets were obtained from the TCGA TARGET GTEX study that includes samples re-analyzed by the same RNA seq pipeline (https://xenabrowser.net/datapages/?dataset=TcgaTargetGtex_RSEM_Hugo_norm_count&host=https%3A%2F%2Ftoil.xenahubs.net&removeHub=https%3A%2F%2Fxena.treehouse.gi.ucsc.edu%3A443, accessed on September 2020). As all samples were processed using a uniform bioinformatic pipeline, the batch effect due to different computational processing was eliminated. TCGA data and data from unrelated healthy tissues, recomputing (RSEM, batch-normalized, log_2_^(x + 1)^-transformed), were downloaded from the UCSC Xena. The TCGA-SKCM (skin cutaneous melanoma) dataset includes 102 primary and 366 metastatic CM samples, while the GTEx dataset contains 556 normal skin samples from healthy donors.

### 5.2. Gene Expression and Promoter Methylation Analysis

*ARL* gene expression in primary, metastatic CM, and normal skin tissue samples collected from TCGA and GTEx, respectively, was displayed in scatter plots performed using GraphPad 8.0.1 software. Gene expression profiles across samples were compared using non-parametric tests, namely the Kruskal–Wallis test with post-hoc Dunn’s method for multiple comparisons. The Gene Expression Profiling Interactive Analysis (GEPIA) database was also used to compare the expression of the 22 *ARL* genes in CM samples and their normal tissue counterparts, as well as their expression in distinct CM TNM stages (http://gepia2.cancer-pku.cn/) [66]. This differential expression analysis was transformed in log2 (transcripts per kilobase million + 1), and one-way ANOVA was used for group comparison [67]. 

To assess whether differences found in *ARL* expression could be related to promoter methylation levels, the UALCAN online tool was used. Therefore, it was used to perform a comprehensive analysis of promoter DNA methylation patterns in normal skin tissue (*n* = 2), primary (*n* = 104), and metastatic CM (*n* = 368) samples. The β-value indicates the level of DNA methylation, ranging from 0 (unmethylated) to 1 (methylated). In this study, β-values ranging from 0.5 to 0.7 indicate hyper-methylation while β-values from 0.25 to 0.3 are suggestive of hypo-methylation [68].

### 5.3. Genomic Analysis

To determine the mutational profile and copy number variation status of *ARL* genes, 367 CM samples from SKCM (TCGA, Firehose Legacy) datasets were uploaded from the cBioportal platform (http://www.cbioportal.org) [69]. In this analysis, we included truncations, missense, and splice mutations (frequency ≤ 1) with unknown clinical significance in CM. The pathogenic effects of these alterations have not been tested yet. The same was verified for amplification and deletions found in these genes.

### 5.4. Gene Ontology Enrichment Analysis

*ARL* expression was subjected to gene ontology (GO) and biological pathway enrichment analyses to elucidate their related biological processes and molecular functions using the Database for Annotation, Visualization and Integrated Discovery (DAVID) (https://david.ncifcrf.gov/summary.jsp) [70], PANTHER 14.0 (http://pantherdb.org) [71,72] and g:Profiler (https://biit.cs.ut.ee) [73] against a *Homo sapiens* background reference. Although all three tools use very similar statistical algorithms in the back end, there are some differences among them. In fact, DAVID downloads PANTHER data and integrates them in its analysis tools. The statistical over-representation was calculated using the binomial test of PANTHER 14.0 and the results were considered significant at *p* < 0.05, after Bonferroni correction. The binomial test is employed to determine whether there is a statistical over-representation of genes in the test list compared to the reference list. This statistical test evaluates whether a specific functional class of genes appears statistically more often in the input list than expected. In the over-representation test, *p*-values were adjusted by default using the Bonferroni correction for multiple testing performed on the PANTHER 14.0 site.

### 5.5. Survival Analysis

Survival data from CM patients were obtained from TCGA, and patients with unavailable clinical information were excluded from this analysis. The correlation between gene expression and overall survival was estimated using the Kaplan–Meier curves and the log-rank test. The Kaplan–-Meier curves were performed using the median expression of each *ARL* gene in normal skin samples as the cutoff to define the groups of CM patients with a high or low *ARL* mRNA expression. The expression thresholds used for each *ARL* gene are shown in Table S12. Variables with a significant *p*-value in the univariable analysis were exposed to a multivariable analysis using the Cox regression proportional hazard model. The multivariable analysis included the following confounder variables: age, gender, tumor grade, type of tumor (primary/metastatic), v-Raf murine sarcoma viral oncogene homolog B1 (*BRAF*), neuroblastoma RAS viral oncogene homolog (*NRAS*) and neurofibromatosis type 1 (*NF1*) mutations and the expression of other *ARL* genes. The *ARL* prognostic value was also assessed using two online survival tools: OncoLnc (http://www.oncolnc.org) and GEPIA [66,74]. In the latter analyses, patients were divided into non-overlapping groups according to the median expression of these genes in CM samples defined using these specific web servers.

### 5.6. Tumor Microenvironment Characterization

The correlation between *ARL* expression and immune cell infiltration was determined using the Tumor Immune Estimation Resource (TIMER) 2.0 server (http://timer.cistrome.org/) [75]. The TIMER platform is frequently used to study the relationship between cancer and immune cell infiltration using several algorithms available for each immune subset. The correlation of *ARL* gene expression with several immune cell infiltrates such as CD8^+^ T lymphocytes, CD4^+^ T lymphocytes, regulatory T cells (Treg), B lymphocytes, neutrophils, monocytes, macrophages, dendritic cells, microglia, Natural Killer (NK) cells, mast cells, cancer-associated fibroblasts, common lymphoid progenitor, common myeloid progenitor, endothelial cell, eosinophil, granulocyte-monocyte progenitor, hematopoietic stem cell, T cell follicular helper, T cell gamma delta, NK T cell, and myeloid-derived suppressor cell tumor was displayed. The correlations between all these immune infiltrate levels and the expression of the 22 *ARL* genes were performed in 3 distinct subsets: SKCM samples, SKCM primary samples, and SKCM-metastatic samples. All these correlations were calculated using the purity (percentage of malignant cells in a tumor tissue) adjustment definition and Spearman’s correlation coefficient. Tumor purity is a major confounding factor in this analysis, since most immune cell types are negatively correlated with tumor purity. Therefore, we selected the “Purity Adjustment” option. Genes highly expressed in cells in the microenvironment are expected to have negative associations with tumor purity, while the opposite is expected for genes highly expressed in the tumor cells. Only genes with Spearman’s coefficient <0.4 were considered positively correlated. 

### 5.7. Protein–Protein Interaction (PPI) Network Analysis

The Search Tool for the Retrieval of Interacting Genes (STRING) (https://string-db.org/) [76] was the database employed to predict ARL functional interactions and their crosstalk with well-known annotated signaling pathways. PPI presents a score that indicates the confidence in the interactions proposed based on the available evidence. This score ranges from 0 to 1, with 1 being the highest possible confidence. Proteins with a score of ≥0.4 were included in the network models visualized. Additionally, the proteins included in PPI networks were divided into a color pattern to distinguish them based on their involvement in specific pathways such as membrane trafficking and vesicular transport, cancer-related pathways, and others.

## Figures and Tables

**Table 1:** Multivariable analysis of *ARL* expression for the overall survival of CM patients available on TCGA.

Variables	*p*-Value	Exp(B)	95.0% CI for Exp(B)	
			Lower	Upper
*ARL1*	0.022	1.642	1.074	2.510
*ARL2*	0.436	1.805	0.408	7.980
*ARL3*	0.094	2.873	0.835	9.882
*ARL4A*	0.120	3.565	0.718	17.705
*ARL4C*	0.211	0.675	0.364	1.250
*ARL4D*	0.952	0.956	0.219	4.163
*ARL5A*	0.561	0.814	0.407	1.628
*ARL5B*	0.513	1.645	0.371	7.306
*ARL5C*	0.720	0.850	0.351	2.063
*ARL6*	0.149	3.268	0.655	16.312
*ARL8A*	0.912	0.955	0.424	2.151
*ARL8B*	0.136	2.144	0.787	5.837
*ARL9*	0.194	4.025	0.492	32.914
*ARL10*	0.413	0.632	0.211	1.895
*ARL11*	0.953	1.080	0.083	14.113
*ARL13A*	0.343	0.561	0.169	1.855
*ARL13B*	0.537	0.801	0.395	1.621
*ARL14*	0.818	0.823	0.157	4.312
*ARL15*	0.009	0.288	0.114	0.730
*ARL16*	0.719	1.243	0.380	4.074
*ARL17A*	0.873	0.801	0.053	12.167
*ARL17B*	0.455	0.395	0.035	4.514
Age	0.000	1.023	1.013	1.034
Gender	0.691	1.070	0.767	1.491
Tumor Grade	0.000	0.200	0.096	0.417
Type of Tumor	0.840	1.239	0.154	9.941
*BRAF* mutations	0.792	1.050	0.732	1.504
*NRAS* mutations	0.402	0.851	0.583	1.242
*NF1* mutations	0.488	1.194	0.723	1.972

## Data Availability

Illumina HiSeq 2000 RNA sequencing data of CM and normal skin tissue samples mentioned in this research are available in the Cancer Genome Atlas (TCGA) and Genotype tissue expression (GTEx) and can also be accessed from the University of California, Santa Cruz (UCSC) Xena project (http://xena.ucsc.edu).

## Supplementary Materials

The following are available online at www.mdpi.com/article/10.3390/ijms22179260/s1.

## Abbreviations

ADAM17	Disintegrin and metalloproteinase domain-containing protein 17
ARF	Adenosine diphosphate–ribosylation factor
ARL	ADP-ribosylation factor-like
BCL2L4	Bcl-2-like protein 14
BRAF	v-raf murine sarcoma viral oncogene homolog B1
CM	Cutaneous melanoma
DAVID	Database for Annotation, Visualization and Integrated Discovery
GO	Gene ontology
GTEx	Genotype tissue expression
HIF1AN	Hypoxia-inducible factor 1-alpha inhibitor
IL17RD	Interleukin-17 receptor D
NF1	Neurofibromatosis type 1
NRAS	Neuroblastoma RAS viral oncogene homolog
P2RX7	P2X purinoceptor 7
NK	Natural killer cells
PPI	Protein–protein interaction
STRING	Search Tool for the Retrieval of Interacting Genes
SKCM	Skin cutaneous melanoma
TCGA	The cancer genome atlas
TIMER	Tumor Immune Estimation Resource
TNM	Tumor, nodes, metastases
TRIM13	E3 ubiquitin–protein ligase
UCSC	University of California, Santa Cruz
GEPIA	Gene Expression Profiling Interactive Analysis
