# Supplementary material for: Unraveling the Relevance of ARL GTPases in Cutaneous Melanoma Prognosis through Integrated Bioinformatics Analysis
Source: Int J Mol Sci. 2021 Aug 26;22(17):9260. doi: 10.3390/ijms22179260 (PMC8431576; doi:10.3390/ijms22179260)
Supplement: Supplementary file 1 [file ijms-22-09260-s001.zip › Tables - major revisions.pdf]

Table S1. Demographics and characteristics of patients with CM downloaded from TCGA.

Patient Characteristics	Classification	Number of Patients Primary CM (n=102)	Number of Patients Metastatic CM (n=366)
**Gender**	Male	60 (58.8 %)	228 (62.3%)
	Female	42 (41.2 %)	138 (37.7%)
**Age at diagnosis (years)**	Median	65	56
**Tumor Stage**	Stage I	2 (2%)	74 (20.2%)
	Stage II	66 (64.7%)	73 (20.0%)
	Stage III	26 (25.5%)	144 (39.3%)
	Stage IV	3 (2.9%)	21 (5.7%)
	Not reported	5 (4.9%)	54 (14.8%)
***BRAF* mutations**	Detected	43 (42.2%)	142 (38.8%)
	Not detected	59 (57.8%)	224 (61.2%)
***NRAS* mutations**	Detected	10 (9.8%)	88 (24%)
	Not detected	92 (90.2%)	278 (76%)
***NF1* mutations**	Detected	9 (8.8%)	37 (10.1%)
	Not detected	93 (91.2%)	329 (89.9%)

Table S2. Multivariable analysis of *ARL1* expression for overall survival of TCGA melanoma patients

	Classification (n)	Mean OS (years)	Standard Error	*p*-value	Exp(B)	95,0% CI for Exp(B)	
						Lower	Upper
**Age**	Mean=59		0,005	0,000	1,021	1,010	1,031
**Gender**	Male (254)		0,159	0,570	1,094	0,802	1,494
	Female (155)						
**Tumor Stage**	Stage I (79)		0,359	0,000	0,258	0,128	0,522
	Stage II (142)						
	Stage III (165)						
	Stage IV (23)						
**Type of tumor**	Primary (97)		0,248	0,002	2,143	1,317	3,487
	Metastatic (312)						
***BRAF* mutations**	Mutated (161)		0,173	0,990	1,002	0,714	1,406
	Wildtype (248)						
***NRAS* mutations**	Mutated (87)		0,184	0,411	0,860	0,599	1,233
	Wildtype (322)						
***NF1* mutations**	Mutated (40)		0,249	0,603	1,139	0,699	1,855
	Wildtype (369)						
ARL1			0,204	0,011	1,676	1,124	2,500
	Low expression (72)	4,8	0,598				
	High expression (337)	11,1	0,738				

Table S3. Multivariable analysis of *ARL3* expression for overall survival of TCGA CM patients.

	Classification (n)	Mean OS (years)	Standard Error	*p*-value	Exp(B)	95,0% CI for Exp(B)	
						Lower	Upper
**Age**	Mean=59		0,005	0,000	1,021	1,011	1,032
**Gender**	Male (254)		0,159	0,457	1,126	0,824	1,537
	Female (155)						
**Tumor Stage**	Stage I (79)		0,357	0,000	0,267	0,132	0,537
	Stage II (142)						
	Stage III (165)						
	Stage IV (23)						
**Type of tumor**	Primary (97)		0,245	0,001	2,334	1,445	3,760
	Metastatic (312)						
***BRAF* mutations**	Mutated (161)		0,172	0,709	1,066	0,761	1,495
	Wildtype (248)						
***NRAS* mutations**	Mutated (87)		0,184	0,471	0,876	0,611	1,256
	Wildtype (322)						
***NF1* mutations**	Mutated (40)		0,248	0,388	1,239	0,761	2,016
	Wildtype (369)						
ARL3			0,171	0,030	0,690	0,493	0,965
	Low expression (96)	11,1					
	High expression (313)	7,3					

Table S4. Multivariable analysis of *ARL5B* expression for overall survival of TCGA melanoma patients.

	Classification (n)	Mean OS (years)	Standard Error	*p*-value	Exp(B)	95,0% CI for Exp(B)	
						Lower	Upper
**Age**	Mean=59		0,005	0,000	1,020	1,010	1,031
**Gender**	Male (254)		0,159	0,512	1,109	0,813	1,513
	Female (155)						
**Tumor Stage**	Stage I (79)		0,357	0,000	0,264	0,131	0,532
	Stage II (142)						
	Stage III (165)						
	Stage IV (23)						
**Type of tumor**	Primary (97)		0,245	0,020	2,160	1,126	4,140
	Metastatic (312)						
***BRAF* mutations**	Mutated (161)		0,172	0,635	1,085	0,774	1,521
	Wildtype (248)						
***NRAS* mutations**	Mutated (87)		0,184	0,534	0,892	0,621	1,280
	Wildtype (322)						
***NF1* mutations**	Mutated (40)		0,248	0,454	1,204	0,741	1,957
	Wildtype (369)						
ARL5B			0,171	0,598	1,143	0,695	1,880
	Low expression (288)	10,8					
	High expression (121)	8,2					

Table S5. Multivariable analysis of *ARL8A* expression for overall survival of TCGA CM patients.

	Classification (n)	Mean OS (years)	Standard Error	*p*-value	Exp(B)	95,0% CI for Exp(B)	
						Lower	Upper
**Age**	Mean=59		0,005	0,000	1,021	1,011	1,031
**Gender**	Male (254)		0,159	0,486	1,117	0,819	1,524
	Female (155)						
**Tumor Stage**	Stage I (79)		0,358	0,000	0,266	0,132	0,535
	Stage II (142)						
	Stage III (165)						
	Stage IV (23)						
**Type of tumor**	Primary (97)		0,322	0,048	1,889	1,006	3,548
	Metastatic (312)						
***BRAF* mutations**	Mutated (161)		0,170	0,678	1,073	0,769	1,499
	Wildtype (248)						
***NRAS* mutations**	Mutated (87)		0,185	0,441	0,867	0,604	1,246
	Wildtype (322)						
***NF1* mutations**	Mutated (40)		0,250	0,372	1,250	0,766	2,041
	Wildtype (369)						
***ARL*8A**			0,331	0,192	0,649	0,340	1,242
	Low expression (65)	4,0					
	High expression (344)	10,6					

Table S6. Multivariable analysis of *ARL10* expression for overall survival of TCGA CM patients.

	Classification (n)	Mean OS (years)	Standard Error	*p*-value	Exp(B)	95,0% CI for Exp(B)	
						Lower	Upper
**Age**	Mean=59		0,005	0,000	1,021	1,011	1,032
**Gender**	Male (254)		0,159	0,548	1,100	0,806	1,501
	Female (155)						
**Tumor Stage**	Stage I (79)		0,357	0,000	0,271	0,135	0,547
	Stage II (142)						
	Stage III (165)						
	Stage IV (23)						
**Type of tumor**	Primary (97)		0,276	0,023	1,875	1,091	3,221
	Metastatic (312)						
***BRAF* mutations**	Mutated (161)		0,172	0,722	1,063	0,759	1,490
	Wildtype (248)						
***NRAS* mutations**	Mutated (87)		0,184	0,490	0,881	0,615	1,262
	Wildtype (322)						
***NF1* mutations**	Mutated (40)		0,249	0,391	1,238	0,760	2,015
	Wildtype (369)						
ARL10			0,190	0,050	1,452	1,283	2,413
	Low expression (263)	11,4					
	High expression (146)	6,7					

Table S7. Multivariable analysis of *ARL11* expression for overall survival of TCGA melanoma patients.

	Classification (n)	Mean OS (years)	Standard Error	*p*-value	Exp(B)	95,0% CI for Exp(B)	
						Lower	Upper
**Age**	Mean=59		0,005	0,000	1,020	1,010	1,031
**Gender**	Male (254)		0,159	0,519	1,108	0,811	1,513
	Female (155)						
**Tumor Stage**	Stage I (79)		0,358	0,000	0,267	0,132	0,538
	Stage II (142)						
	Stage III (165)						
	Stage IV (23)						
**Type of tumor**	Primary (97)		1,020	0,427	2,248	0,304	16,603
	Metastatic (312)						
***BRAF* mutations**	Mutated (161)		0,171	0,677	1,074	0,768	1,502
	Wildtype (248)						
***NRAS* mutations**	Mutated (87)		0,184	0,510	0,866	0,618	1,270
	Wildtype (322)						
***NF1* mutations**	Mutated (40)		0,248	0,452	1,205	0,742	1,958
	Wildtype (369)						
ARL11			1,035	0,936	0,920	0,121	6,995
	Low expression (95)	2,8					
	High expression (314)	10,7					

Table S8. Multivariable analysis of *ARL13A* expression for overall survival of TCGA melanoma patients.

	Classification (n)	Mean OS (years)	Standard Error	*p*-value	Exp(B)	95,0% CI for Exp(B)	
						Lower	Upper
**Age**	Mean=59		0,005	0,000	1,022	1,011	1,032
**Gender**	Male (254)		0,159	0,619	1,082	0,792	1,478
	Female (155)						
**Tumor Stage**	Stage I (79)		0,359	0,000	0,250	0,124	0,506
	Stage II (142)						
	Stage III (165)						
	Stage IV (23)						
**Type of tumor**	Primary (97)		0,254	0,000	2,561	1,583	4,143
	Metastatic (312)						
***BRAF* mutations**	Mutated (161)		0,172	0,849	1,033	0,738	1,447
	Wildtype (248)						
***NRAS* mutations**	Mutated (87)		0,185	0,427	0,864	0,601	1,241
	Wildtype (322)						
***NF1* mutations**	Mutated (40)		0,248	0,392	1,237	0,760	2,012
	Wildtype (369)						
ARL13A			0,278	0,029	0,544	0,316	0,938
	Low expression (384)	10,6					
	High expression (25)	5,5					

Table S9. Multivariable analysis of *ARL15* expression for overall survival of TCGA melanoma patients.

	Classification (n)	Mean OS (years)	Standard Error	*p*-value	Exp(B)	95,0% CI for Exp(B)	
						Lower	Upper
**Age**	Mean=59		0,005	0,000	1,021	1,011	1,032
**Gender**	Male (254)		0,159	0,440	1,131	0,828	1,544
	Female (155)						
**Tumor Stage**	Stage I (79)		0,357	0,000	0,262	0,130	0,528
	Stage II (142)						
	Stage III (165)						
	Stage IV (23)						
**Type of tumor**	Primary (97)		0,822	0,001	2,275	1,407	3,680
	Metastatic (312)						
***BRAF* mutations**	Mutated (161)		0,163	0,713	1,065	0,760	1,494
	Wildtype (248)						
***NRAS* mutations**	Mutated (87)		0,138	0,452	0,871	0,607	1,249
	Wildtype (322)						
***NF1* mutations**	Mutated (40)		0,224	0,368	1,251	0,768	2,037
	Wildtype (369)						
ARL15			0,494	0,003	0,610	0,443	0,841
	Low expression (297)	10,3					
	High expression (112)	6,8					

Table S10. Multivariable analysis of *ARL16* expression for overall survival of TCGA melanoma patients.

	Classification (n)	Mean OS (years)	Standard Error	*p*-value	Exp(B)	95,0% CI for Exp(B)	
						Lower	Upper
**Age**	Mean=59		0,005	0,000	1,021	1,011	1,032
**Gender**	Male (254)		0,159	0,525	1,106	0,810	1,510
	Female (155)						
**Tumor Stage**	Stage I (79)		0,357	0,000	0,271	0,135	0,546
	Stage II (142)						
	Stage III (165)						
	Stage IV (23)						
**Type of tumor**	Primary (97)		0,327	0,101	1,709	0,900	3,247
	Metastatic (312)						
***BRAF* mutations**	Mutated (161)		0,171	0,728	1,061	0,759	1,485
	Wildtype (248)						
***NRAS* mutations**	Mutated (87)		0,183	0,501	0,884	0,617	1,266
	Wildtype (322)						
***NF1* mutations**	Mutated (40)		0,249	0,361	1,256	0,771	2,047
	Wildtype (369)						
ARL16			0,256	0,110	0,665	0,402	1,098
	Low expression (282)	11,2					
	High expression (127)	4,9					

Table S11. Correlation between *ARL* genes expression and the infiltration of distinct immune cell subsets, in SKCM, primary and metastatic melanoma samples. Data obtained from the TIMER2.0 resource, including *p*-values and Spearman's rank correlation coefficients (cor) according to different algorithms applied. Only significant correlations were presented in the table.

Gene	Description	Algorithm	SKCM		SKCM - Primary		SKCM - Metastasis	
			cor	*p*-value	cor	*p*-value	cor	*p*-value
ARL1	T cells CD4+ memory	XCELL	0.447	8.81×10^{-24}	0.725	6.76×10^{-18}	0.3	8.78×10^{-09}
	T cells CD4+ Th1	XCELL	-0.577	5.60×10^{-42}	-0.601	2.39×10^{-42}	-0.568	1.41×10^{-31}
	T cells CD4+ Th2	XCELL	0.261	1.56×10^{-08}	0.598	3.11×10^{-11}	0.098	6.68×10^{-02}
	Treg	QUANTISEQ	0.351	1.06×10^{-14}	0.458	1.31×10^{-06}	0.278	1.11×10^{-07}
	Neutrophils	TIMER	0.427	1.17×10^{-21}	0.501	8.17×10^{-08}	0.349	1.38×10^{-11}
	Common lymphoid progenitor	XCELL	0.485	2.25×10^{-28}	0.72	1.53×10^{-17}	0.411	7.87×10^{-16}
ARL2	T cells CD4+ Th1	XCELL	0.369	3.76×10^{-16}	0.52	4.80×10^{-09}	0.312	2.04×10^{-09}
ARL3	T cells CD8+	TIMER	0.231	5.80×10^{-07}	0.47	6.02×10^{-07}	0.133	1.25×10^{-02}
	T cells CD4+ memory	XCELL	0.348	2.01×10^{-14}	0.502	7.86×10^{-08}	0.263	5.35×10^{-07}
	T cells CD4+ Th2	XCELL	0.321	2.03×10^{-12}	0.47	6.29×10^{-07}	0.243	3.88×10^{-06}
	Common lymphoid progenitor	XCELL	0.325	1.02×10^{-12}	0.451	1.96×10^{-06}	0.287	3.89×10^{-08}
ARL4A	Common lymphoid progenitor	XCELL	0.28	1.05×10^{-09}	0.433	5.37×10^{-06}	0.23	1.25×10^{-05}
ARL4C	T cells CD4+ memory	XCELL	0.444	1.51×10^{-23}	0.364	1.43×10^{-4}	0.384	7.28×10^{-14}
	T cells CD4+ Th2	XCELL	0.307	2.06×10^{-11}	0.409	2.01×10^{-5}	0.22	3.10×10^{-05}
	Treg	QUANTISEQ	0.409	7.43×10^{-20}	0.363	1.79×10^{-04}	0.376	2.36×10^{-13}
	Neutrophils	TIMER	0.508	2.34×10^{-31}	0.362	1.87×10^{-04}	0.517	1.22×10^{-25}
	Macrophages M2	CIBERSORT-ABS	0.433	2.79×10^{-22}	0.363	1.80×10^{-04}	0.407	1.56×10^{-15}
	Monocyte	MCP-COUNTER	0.571	5.77×10^{-41}	0.321	1.01×10^{-03}	0.581	2.34×10^{-33}
ARL5A	T cells CD4+	EPIC	0.46	2.70×10^{-25}	0.595	4.09×10^{-11}	0.421	1.18×10^{-16}
	T cells CD4+ memory	XCELL	0.57	9.75×10^{-41}	0.603	1.95×10^{-11}	0.525	1.95×10^{-11}
	T cells CD4+ Th2	XCELL	0.388	7.87×10^{-18}	0.459	$1.21\times^{10-06}$	0.32	6.88×10^{-10}
	Neutrophils	TIMER	0.556	1.88×10^{-38}	0.602	2.25×10^{-11}	0.508	1.19×10^{-24}

		MCP-COUNTER	0.405	1.93×10^{-19}	0.568	4.82×10^{-10}	0.377	2.25×10^{-13}
	**Common lymphoid progenitor**	XCELL	0.52	5.02×10^{-33}	0.657	6.23×10^{-14}	0.48	9.20×10^{-22}
ARL5B	**T cells CD4+**	EPIC	0.413	3.29×10^{-20}	0.402	2.78×10^{-05}	0.418	2.08×10^{-16}
	**T cells CD4+ memory**	XCELL	0.552	7.11×10^{-38}	0.679	4.43×10^{-15}	0.439	4.20×10^{-18}
	**T cells CD4+ Th2**	XCELL	0.417	1.12×10^{-20}	0.557	1.19×10^{-09}	0.315	1.28×10^{-09}
	**Neutrophils**	TIMER	0.51	1.46×10^{-31}	0.537	6.01×10^{-09}	0.447	8.39×10^{-19}
		MCP-COUNTER	0.4	5.99×10^{-19}	0.395	4.04×10^{-05}	0.431	1.90×10^{-17}
	**Common lymphoid progenitor**	XCELL	0.495	1.16×10^{-29}	0.644	2.82×10^{-13}	0.467	1.31×10^{-20}
	**T cell NK**	XCELL	-0.417	1.17×10^{-20}	-0.581	1.53×10^{-10}	-0.355	5.63×10^{-12}
ARL6	**T cells CD4+ memory**	XCELL	0.465	6.03×10^{-26}	0.679	4.34×10^{-15}	0.308	3.05×10^{-09}
	**T cells CD4+ Th1**	XCELL	-0.499	4.27×10^{-30}	-0.532	8.65×10^{-09}	-0.495	2.77×10^{-23}
	**T cells CD4+ Th2**	XCELL	0.266	7.76×10^{-09}	0.525	1.50×10^{-08}	0.12	2.41×10^{-02}
	**Treg**	QUANTISEQ	0.385	1.30×10^{-17}	0.411	1.76×10^{-05}	0.323	4.85×10^{-10}
	**Neutrophils**	TIMER	0.481	8.67×10^{-28}	0.519	2.23×10^{-08}	0.407	1.53×10^{-15}
		MCP-COUNTER	0.422	3.66×10^{-21}	0.388	5.51×10^{-05}	0.462	4.22×10^{-20}
	**Common lymphoid progenitor**	XCELL	0.577	6.02×10^{-42}	0.703	1.74×10^{-16}	0.543	1.67×10^{-28}
	**T cell NK**	XCELL	-0.419	6.80×10^{-21}	-0.554	1.56×10^{-09}	-0.365	1.32×10^{-12}
ARL8B	**Neutrophils**	TIMER	0.435	1.74×10^{-22}	0.548	2.44×10^{-09}	0.381	1.15×10^{-13}
		MCP-COUNTER	0.386	1.13×10^{-17}	0.48	3.23×10^{-07}	0.365	1.38×10^{-12}
	**Common lymphoid progenitor**	XCELL	0.413	2.70×10^{-20}	0.642	3.74×10^{-13}	0.337	7.5×10^{-11}
	**T cell NK**	XCELL	-0.228	8.43×10^{-07}	-0.484	2.61×10^{-07}	-0.135	1.12×10^{-02}
ARL11	**T cells CD8+**	EPIC	0.281	9.52×10^{-10}	0.469	6.68×10^{-07}	0.229	1.33×10^{-05}
		MCP-COUNTER	0.593	9.16×10^{-45}	0.373	1.14×10^{-04}	0.575	1.42×10^{-32}
		QUANTISEQ	0.571	6.75×10^{-41}	0.305	1.81×10^{-03}	0.565	3.41×10^{-31}
		CIBERSORT-ABS	0.576	9.37×10^{-42}	0.348	3.44×10^{-04}	0.576	1.18×10^{-32}
		XCELL	0.489	7.12×10^{-29}	0.376	9.88×10^{-05}	0.477	1.81×10^{-21}
	**T cells CD8+ central memory**	XCELL	0.572	5.16×10^{-41}	0.368	1.41×10^{-04}	0.564	3.83×10^{-31}
	**T cells CD4+ memory**	XCELL	0.602	2.48×10^{-46}	0.485	2.47×10^{-07}	0.542	1.87×10^{-28}
	**B cell**	QUANTISEQ	0.404	2.41×10^{-19}	0.242	1.44×10^{-02}	0.341	4.32×10^{-11}
		XCELL	0.498	4.50×10^{-30}	0.32	1.05×10^{-03}	0.434	1.18×10^{-17}

		MCP-COUNTER	0.443	2.46×10^{-23}	0.334	6.13×10^{-04}	0.359	3.47×10^{-12}
	**Memory B cell**	TIMER	0.463	1.03×10^{-25}	0.435	4.79×10^{-06}	0.387	4.36×10^{-14}
	**Neutrophil**	TIMER	0.697	8.74×10^{-68}	0.666	2.19×10^{-14}	0.667	5.93×10^{-47}
	**Macrophage**	EPIC	0.551	1.20×10^{-37}	0.327	7.93×10^{-04}	0.568	1.33×10^{-31}
		XCELL	0.603	1.58×10^{-46}	0.412	1.65×10^{-05}	0.573	2.58×10^{-32}
	**Macrophage M1**	CIBERSORT-ABS	0.612	2.28×10^{-48}	0.472	5.40×10^{-07}	0.583	1.49×10^{-33}
		QUANTISEQ	0.462	1.64×10^{-25}	0.377	9.38×10^{-05}	0.414	4.44×10^{-16}
		XCELL	0.662	6.25×10^{-59}	0.486	2.25×10^{-07}	0.64	2.89×10^{-42}
	**Macrophage M2**	CIBERSORT-ABS	0.739	5.21×10^{-80}	0.692	8.48×10^{-16}	0.718	2.71×10^{-57}
		QUANTISEQ	0.517	1.30×10^{-32}	0.225	2.27×10^{-02}	0.553	9.55×10^{-30}
		TIDE	-0.604	1.01×10^{-46}	-0.545	3.26×10^{-09}	-0.57	6.24×10^{-32}
	**Monocyte**	MCP-COUNTER	0.842	4.32×10^{-124}	0.863	2.41×10^{-31}	0.809	3.06×10^{-83}
		XCELL	0.605	4.92×10^{-47}	0.37	1.29×10^{-04}	0.647	2.19×10^{-43}
	**Myeloid Dendritic Cell**	TIMER	0.514	3.59×10^{-32}	0.275	5.13×10^{-03}	0.547	5.82×10^{-29}
		XCELL	0.429	6.58×10^{-22}	0.305	1.84×10^{-03}	0.465	2.08×10^{-20}
		MCP-COUNTER	0.624	1.29×10^{-50}	0.49	1.71×10^{-07}	0.614	5.24×10^{-38}
	**Myeloid Dendritic Cell Activated**	XCELL	0.612	2.42×10^{-48}	0.415	1.42×10^{-05}	0.585	7.72×10^{-34}
	**Plasmocytoid Dendritic Cell**	XCELL	0.496	9.91×10^{-30}	0.211	3.36×10^{-02}	0.519	7.82×10^{-26}
	**NK cell**	MCP-COUNTER	0.546	8.13×10^{-37}	0.433	5.62×10^{-06}	0.547	5.52×10^{-29}
	**NK cell Actvated**	MCP-COUNTER	0.467	4.14×10^{-26}	0.454	1.68×10^{-06}	0.445	1.26×10^{-18}
	**T cell follicular helper**	CIBERSORT-ABS	0.508	2.23×10^{-31}	0.42	1.14×10^{-05}	0.515	2.17×10^{-25}
ARL13B	**T cells CD4+ memory**	XCELL	0.522	2.68×10^{-33}	0.662	3.37×10^{-14}	0.428	3.27×10^{-17}
	**T cells CD4+ Th2**	XCELL	0.36	1.92×10^{-15}	0.534	7.35×10^{-09}	0.259	7.97×10^{-07}
	**Treg**	QUANTISEQ	0.463	1.24×10^{-25}	0.45	2.05×10^{-06}	0.431	2.04×10^{-17}
	**Neutrophil**	MCP-COUNTER	0.449	4.60×10^{-24}	0.452	1.89×10^{-06}	0.478	1.21×10^{-21}
		TIMER	0.545	9.86×10^{-37}	0.5	8.62×10^{-08}	0.518	9.65×10^{-26}
	**Common lymphoid progenitor**	XCELL	0.54	5.49×10^{-36}	0.77	3.23×10^{-21}	0.471	6.04×10^{-21}

	**T cell NK**	XCELL	-0.428	8.81×10^{-22}	-0.601	2.44×10^{-11}	-0.361	2.29×10-12
ARL15	**T cell CD4+**	EPIC	0.431	4.43×10^{-22}	0.542	4.06×10^{-09}	0.393	1.72×10^{-14}
	**T cell CD4+ memory**	XCELL	0.419	7.47×10^{-21}	0.353	2.71×10^{-04}	0.36	3.01×10^{-12}
	**T cells CD4+ Th1**	XCELL	-0.506	4.26×10^{-31}	-0.541	4.20×10^{-09}	-0.5	8.59×10^{-24}
	**Tregs**	QUANTISEQ	0.392	3.29×10^{-18}	0.446	2.70×10^{-06}	0.342	3.70×10^{-11}
	**Neutrophil**	TIMER	0.559	6.21×10^{-39}	0.6	2.62×10^{-11}	0.511	5.58×10^{-25}
	**Macrophage M2**	CIBERSORT-ABS	0.504	7.57×10^{-31}	0.409	1.99×10^{-05}	0.515	2.37×10^{-25}
	**Monocytes**	QUANTISEQ	0.344	4.11×10^{-14}	0.477	4.02×10^{-07}	0.339	5.44×10^{-11}
	**Myeloid Dendritic cell**	MCP-COUNTER	0.444	1.68×10^{-23}	0.428	7.14×10^{-06}	0.403	2.94×10^{-15}
	**NK cell**	QUANTISEQ	0.405	1.98×10^{-19}	0.448	2.37×10^{-06}	0.424	7.05×10^{-17}
	**T cell NK**	XCELL	-0.383	1.96×10^{-17}	-0.541	4.43×10^{-09}	-0.31	2.57×10^{-09}

Table S12. Expression thresholds defined to divide patients into two non-overlapping groups with high or low *ARL* expression

Variables	Threshold
ARL1	10,72
ARL2	11.09
ARL3	9.50
ARL4A	11.125
ARL4C	10.43
ARL4D	10.25
ARL5A	11.04
ARL5B	10.03
ARL5C	1.13
ARL6	7.44
ARL8A	10.62
ARL8B	12.26
ARL9	4.80
ARL10	9.57
ARL11	4.27
ARL13A	1.70
ARL13B	8.01
ARL14	1.29
ARL15	9.54
ARL16	9.88
ARL17A	9.99
ARL17B	6.75
