# Supplementary figures and images for: Unraveling the Relevance of ARL GTPases in Cutaneous Melanoma Prognosis through Integrated Bioinformatics Analysis
Source: Int J Mol Sci. 2021 Aug 26;22(17):9260. doi: 10.3390/ijms22179260 (PMC8431576; doi:10.3390/ijms22179260)

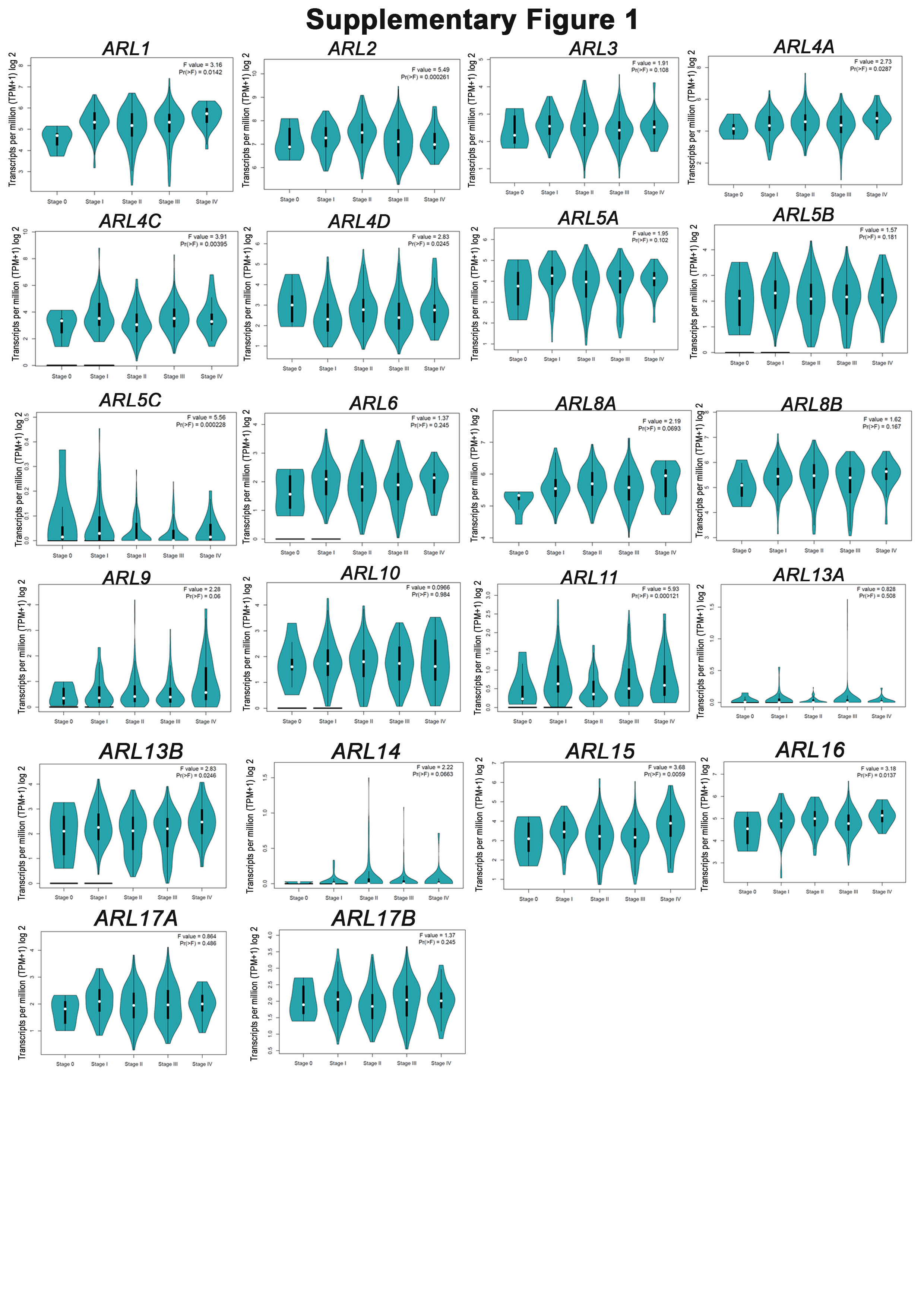

Supplement: Supplementary file 1 [file ijms-22-09260-s001.zip › Supplementary Figure S1.jpg]

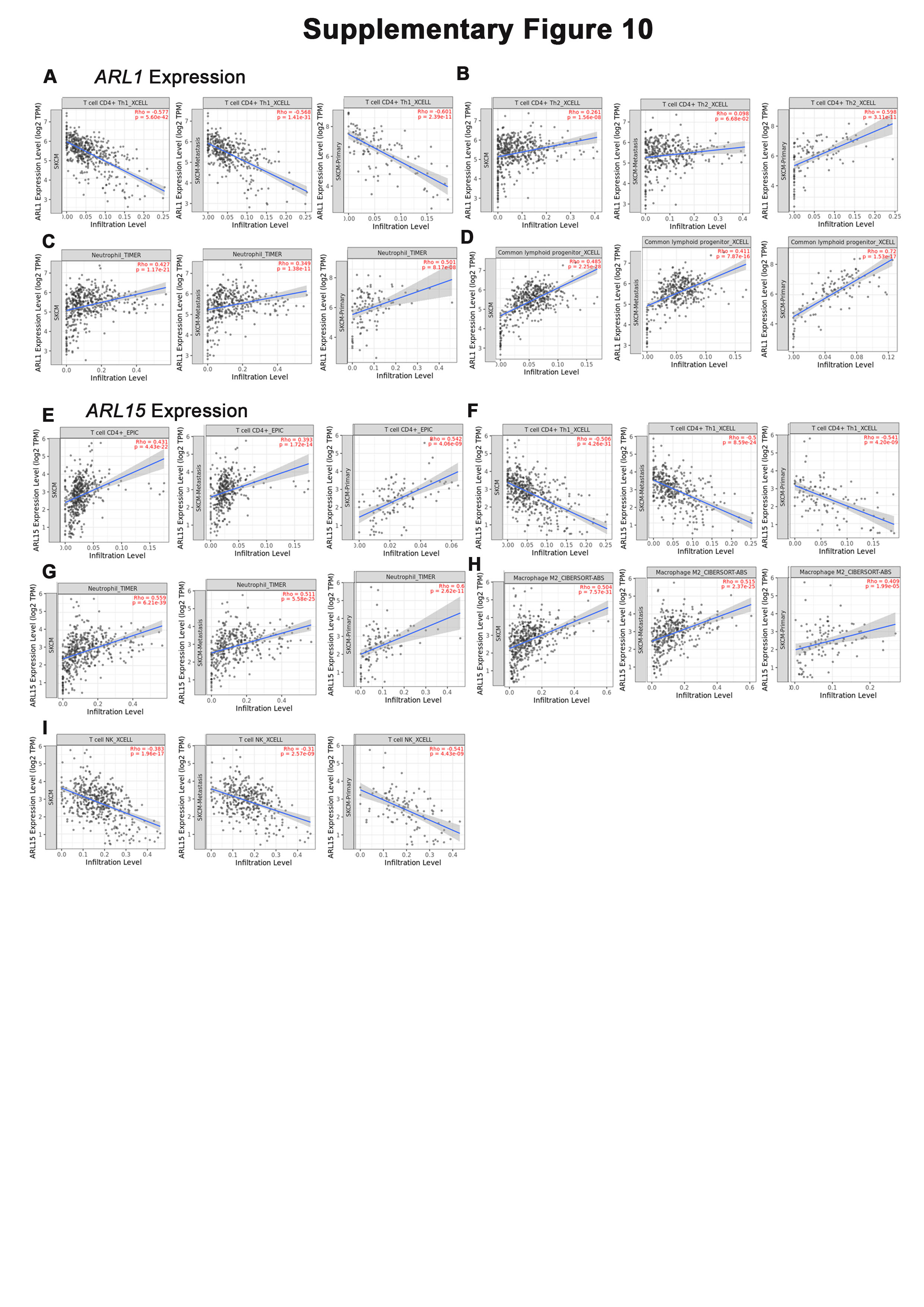

Supplement: Supplementary file 1 [file ijms-22-09260-s001.zip › Supplementary Figure S10.jpg]

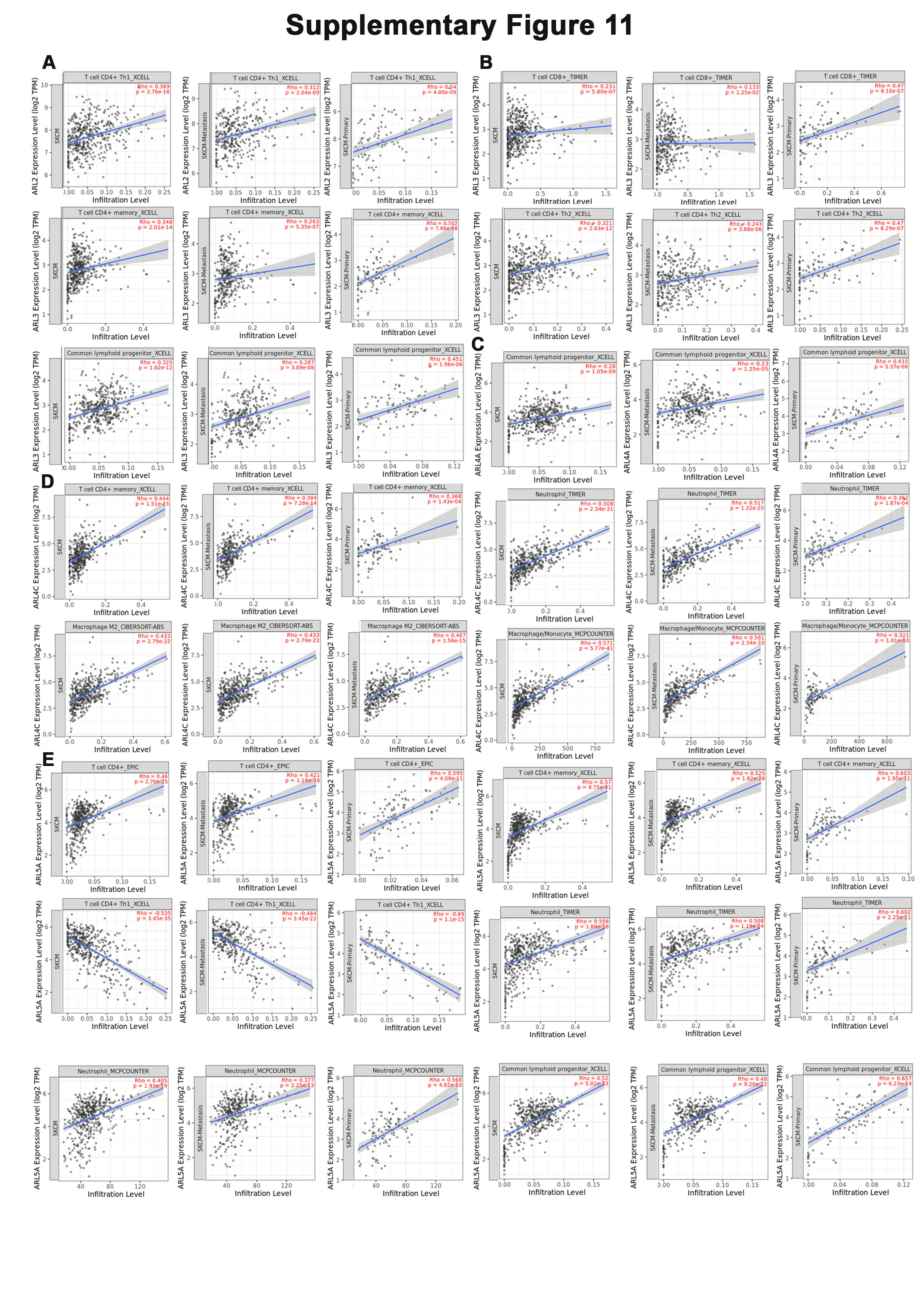

Supplement: Supplementary file 1 [file ijms-22-09260-s001.zip › Supplementary Figure S11.jpg]

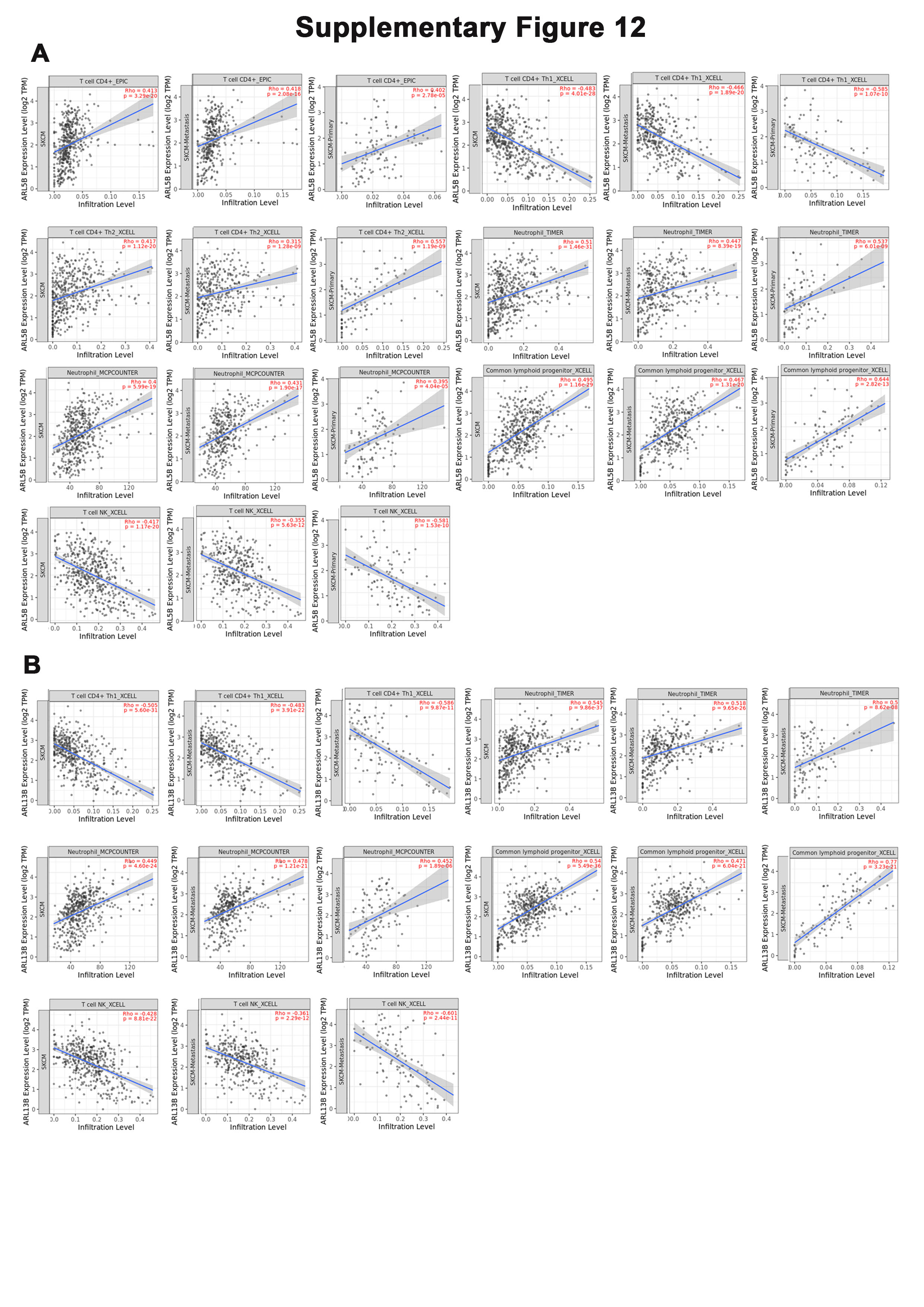

Supplement: Supplementary file 1 [file ijms-22-09260-s001.zip › Supplementary Figure S12.jpg]

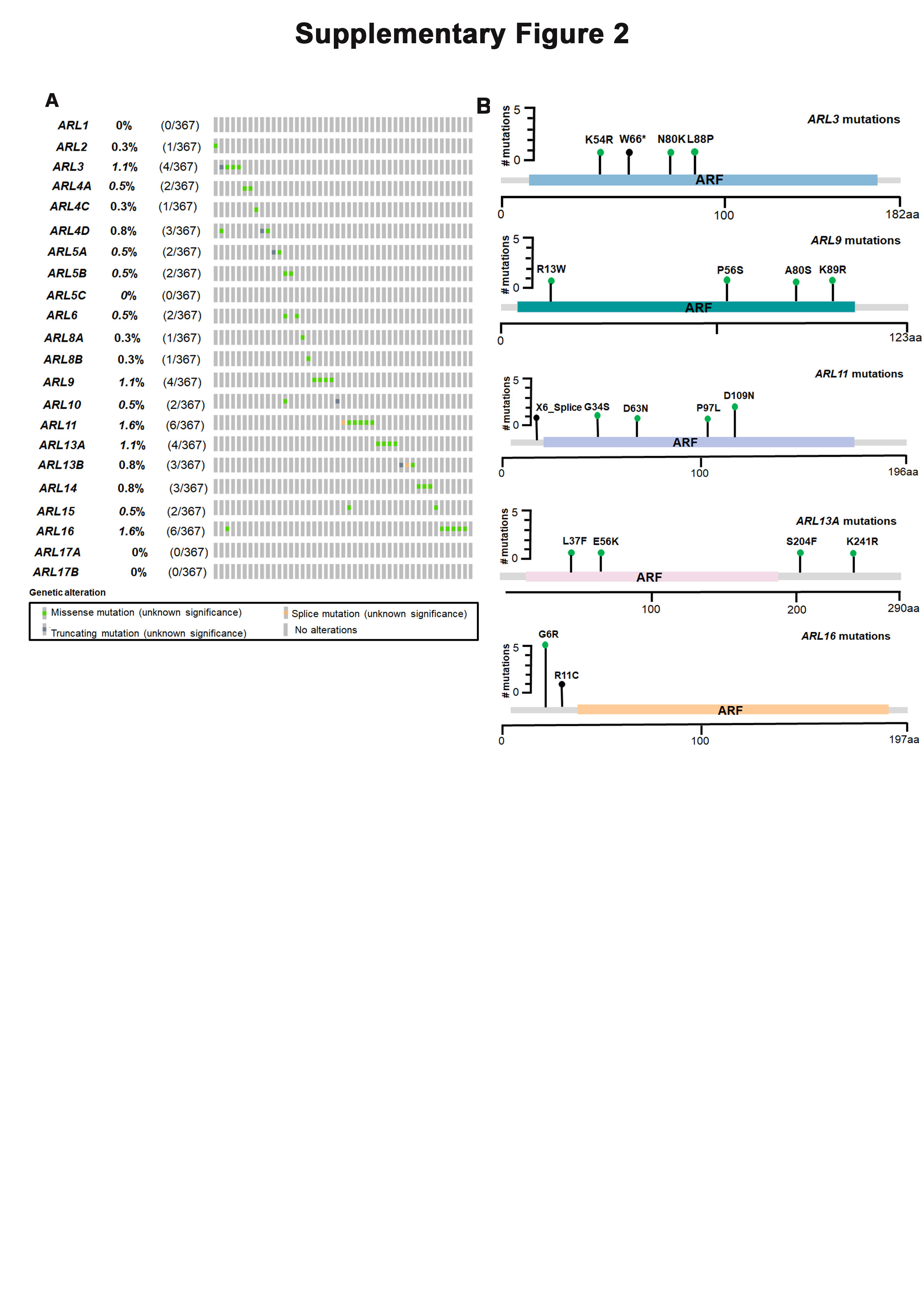

Supplement: Supplementary file 1 [file ijms-22-09260-s001.zip › Supplementary Figure S2.jpg]

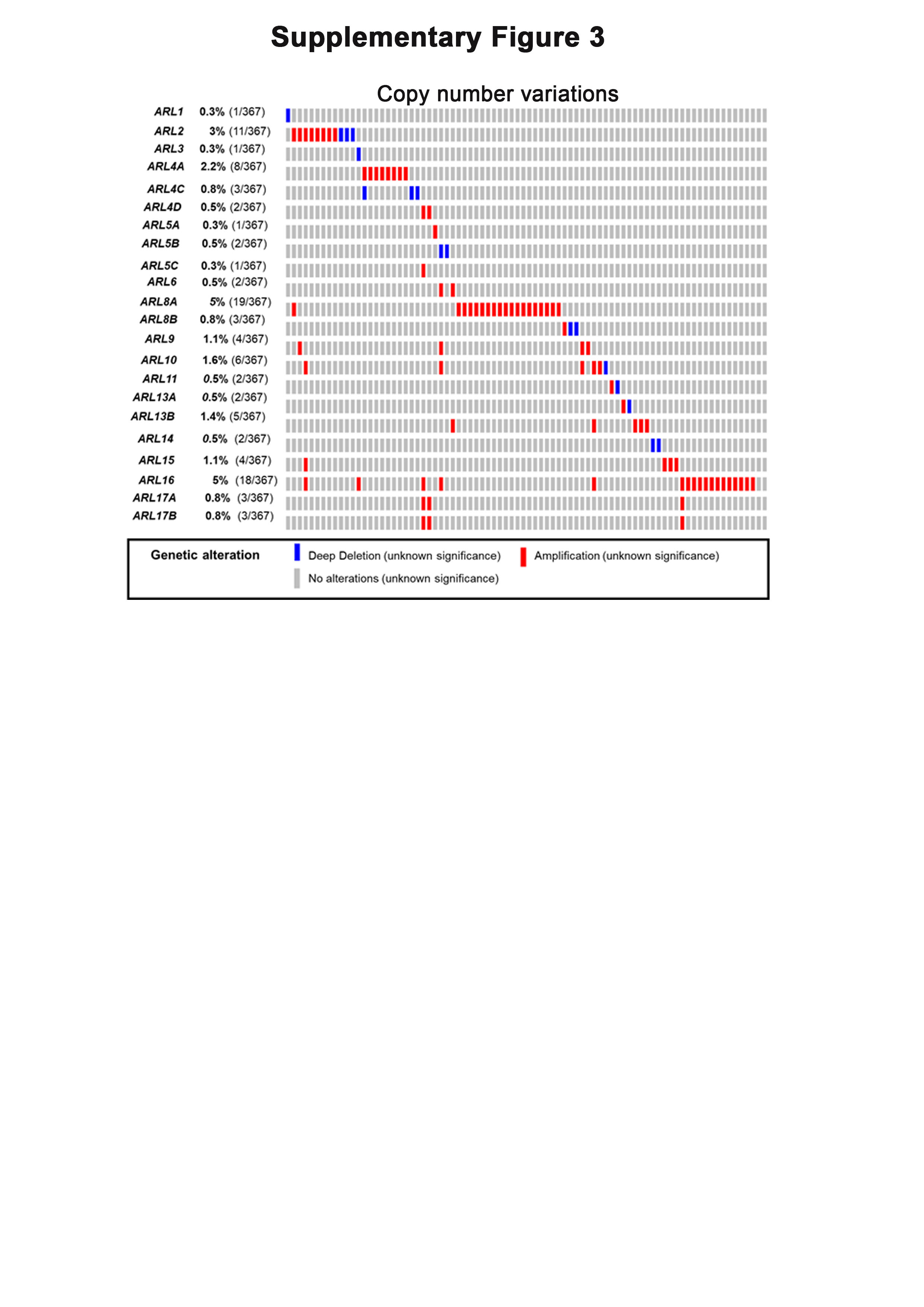

Supplement: Supplementary file 1 [file ijms-22-09260-s001.zip › Supplementary Figure S3.jpg]

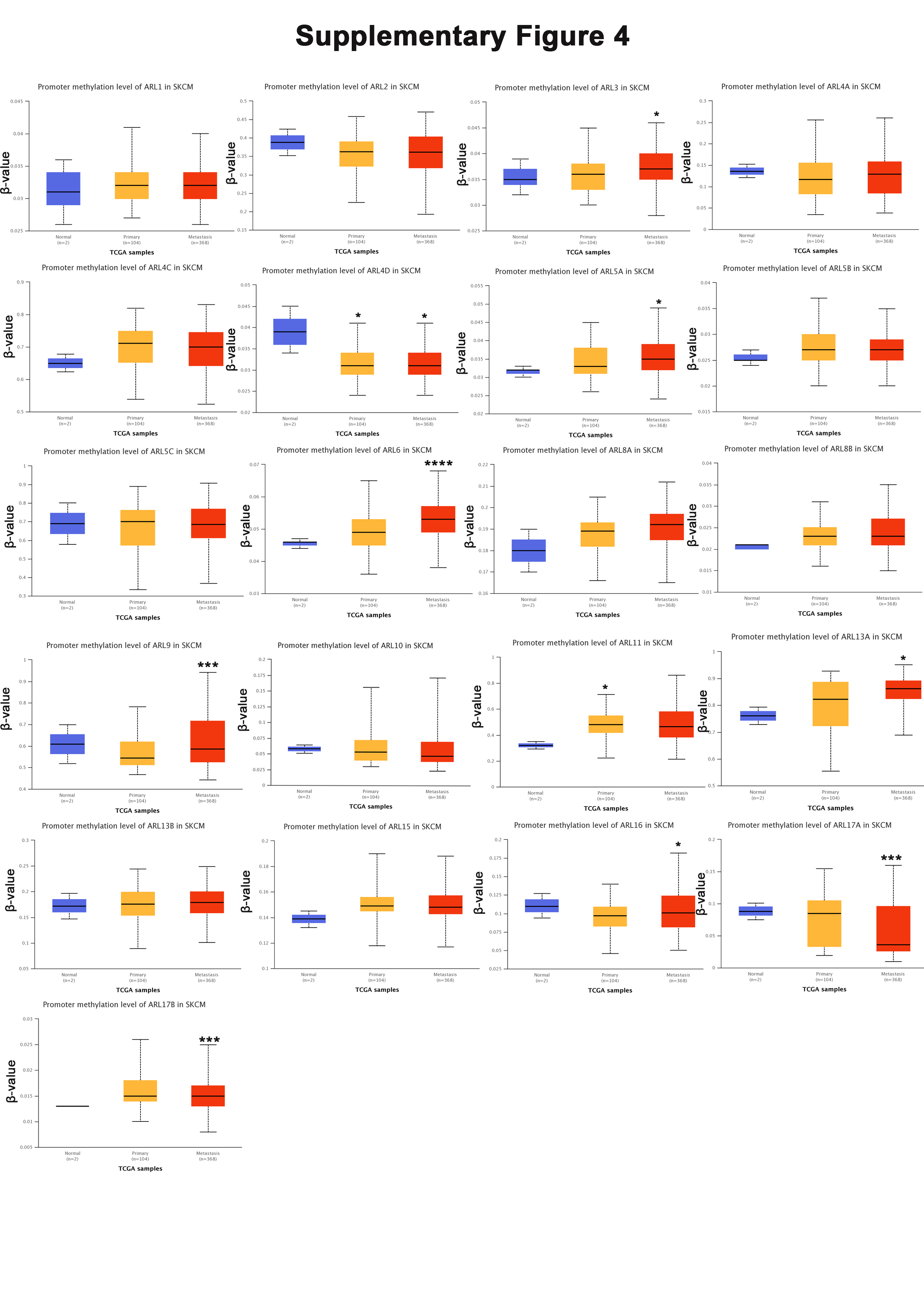

Supplement: Supplementary file 1 [file ijms-22-09260-s001.zip › Supplementary Figure S4.tif]

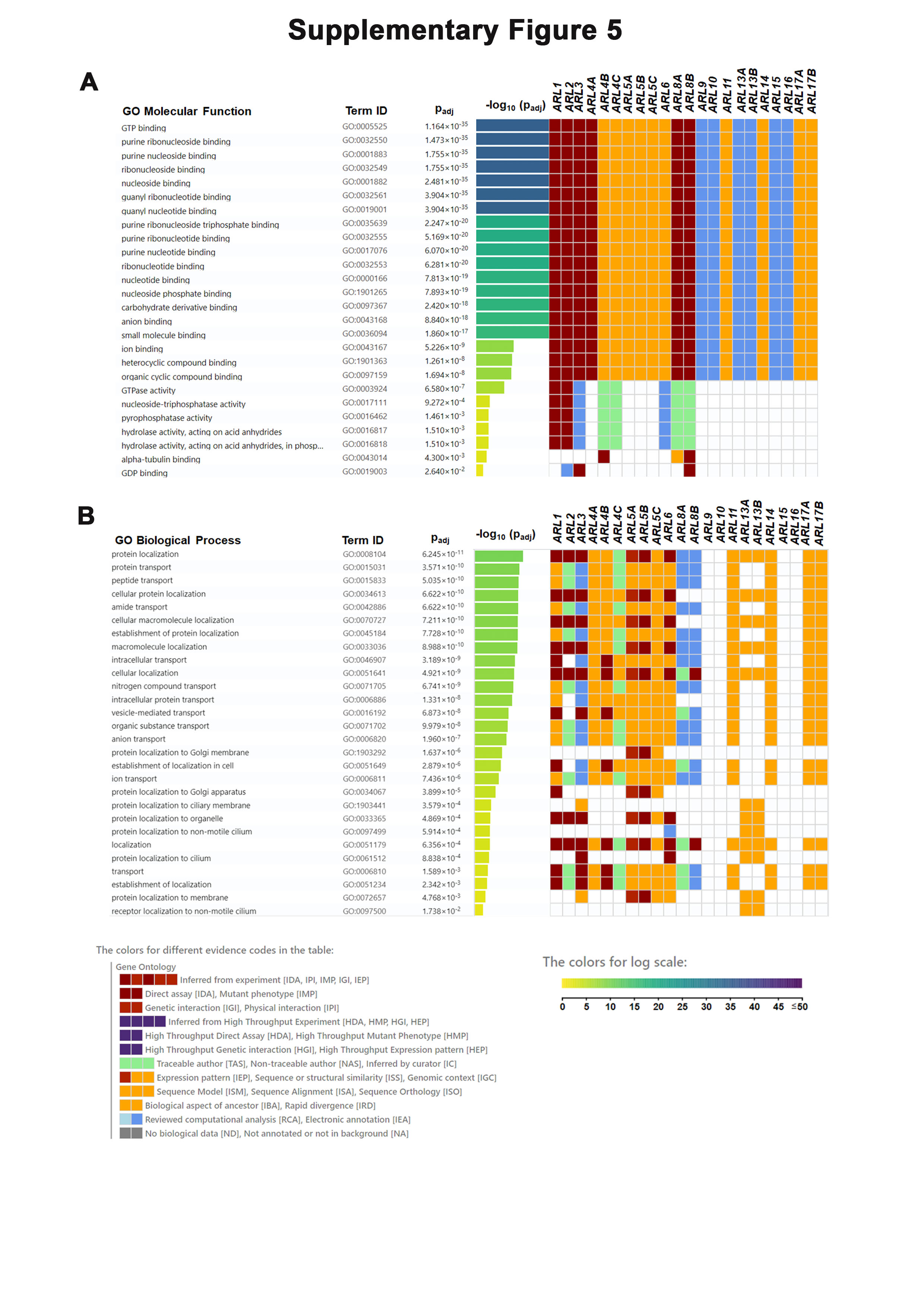

Supplement: Supplementary file 1 [file ijms-22-09260-s001.zip › Supplementary Figure S5.jpg]

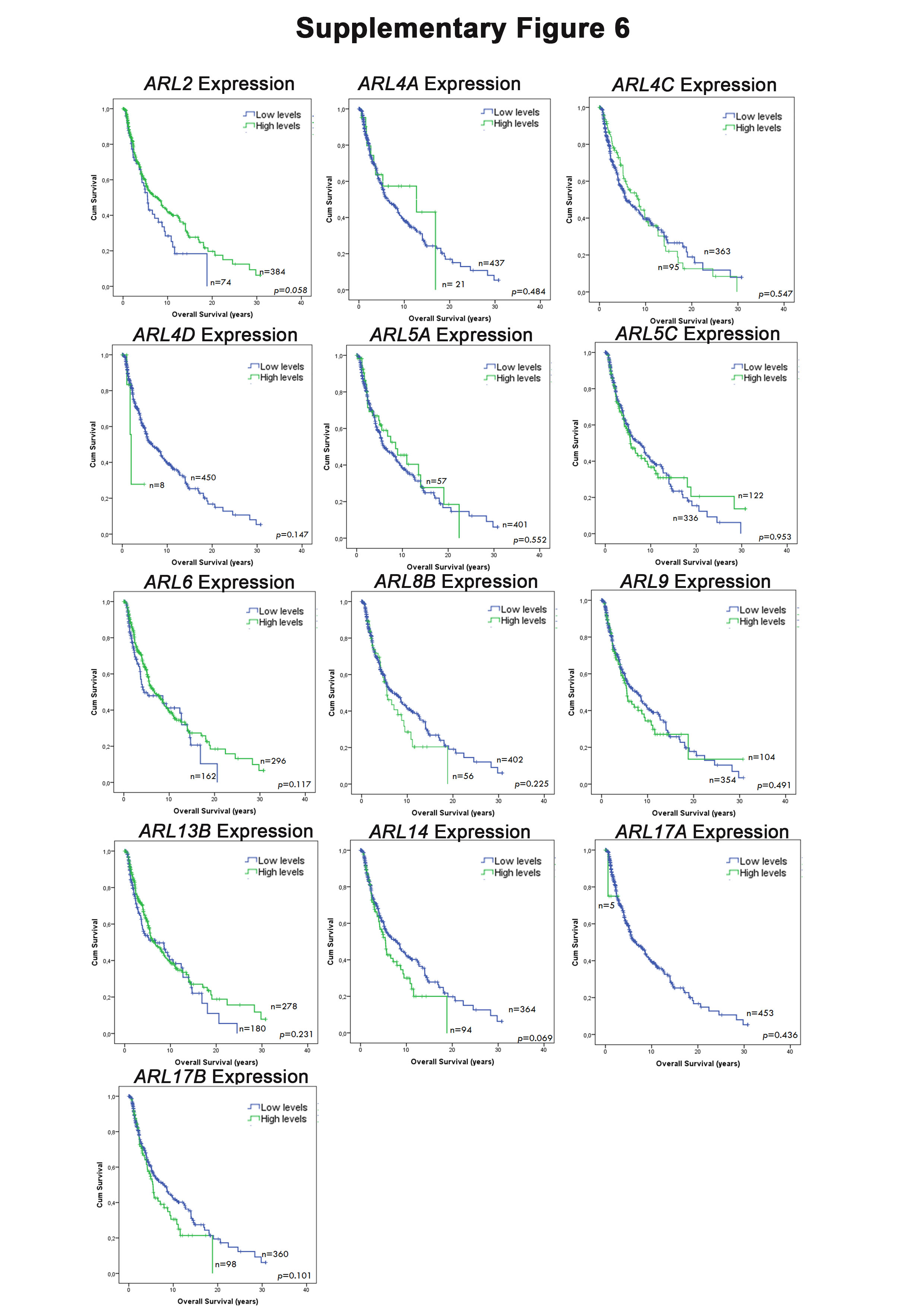

Supplement: Supplementary file 1 [file ijms-22-09260-s001.zip › Supplementary Figure S6.jpg]

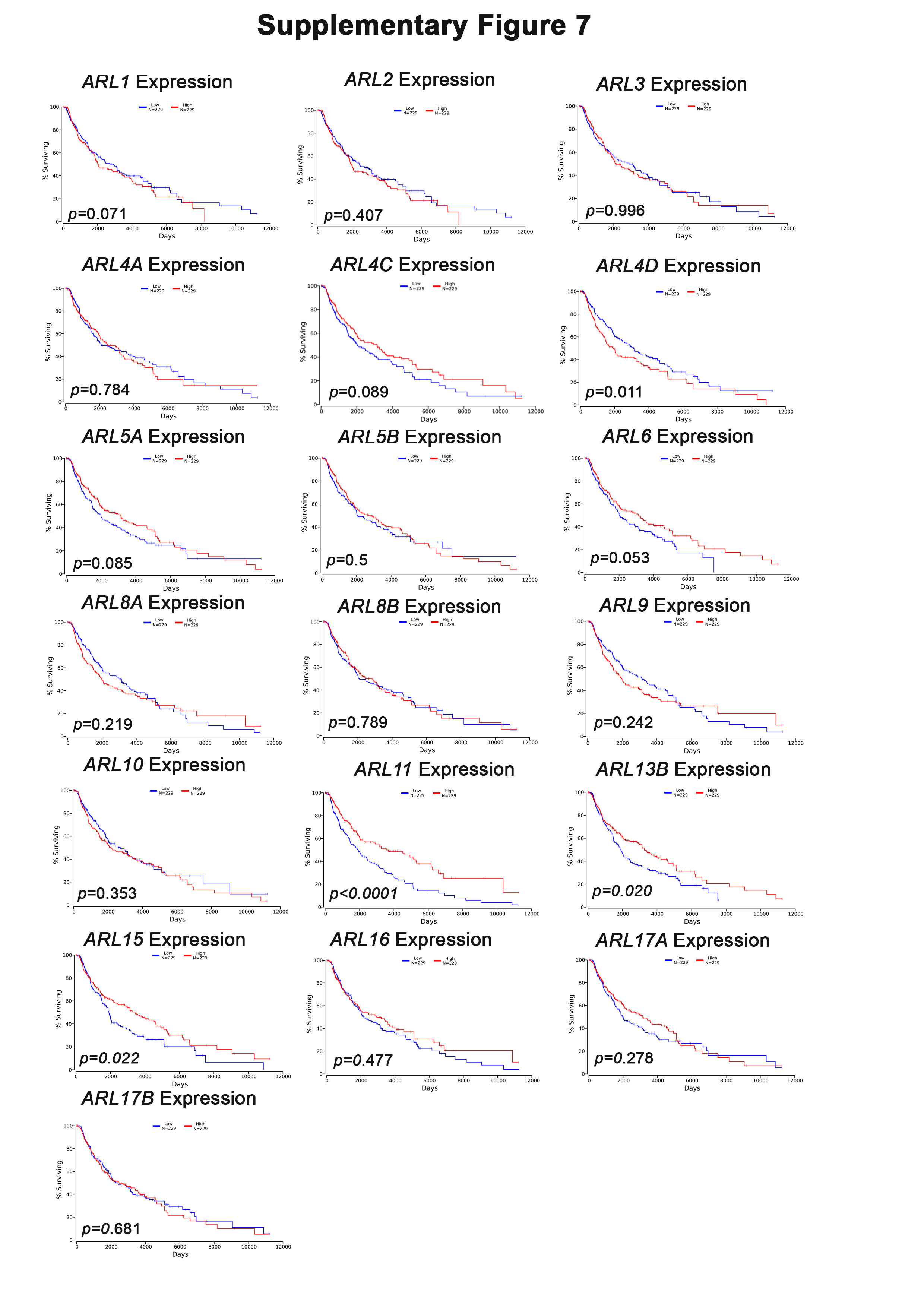

Supplement: Supplementary file 1 [file ijms-22-09260-s001.zip › Supplementary Figure S7.jpg]

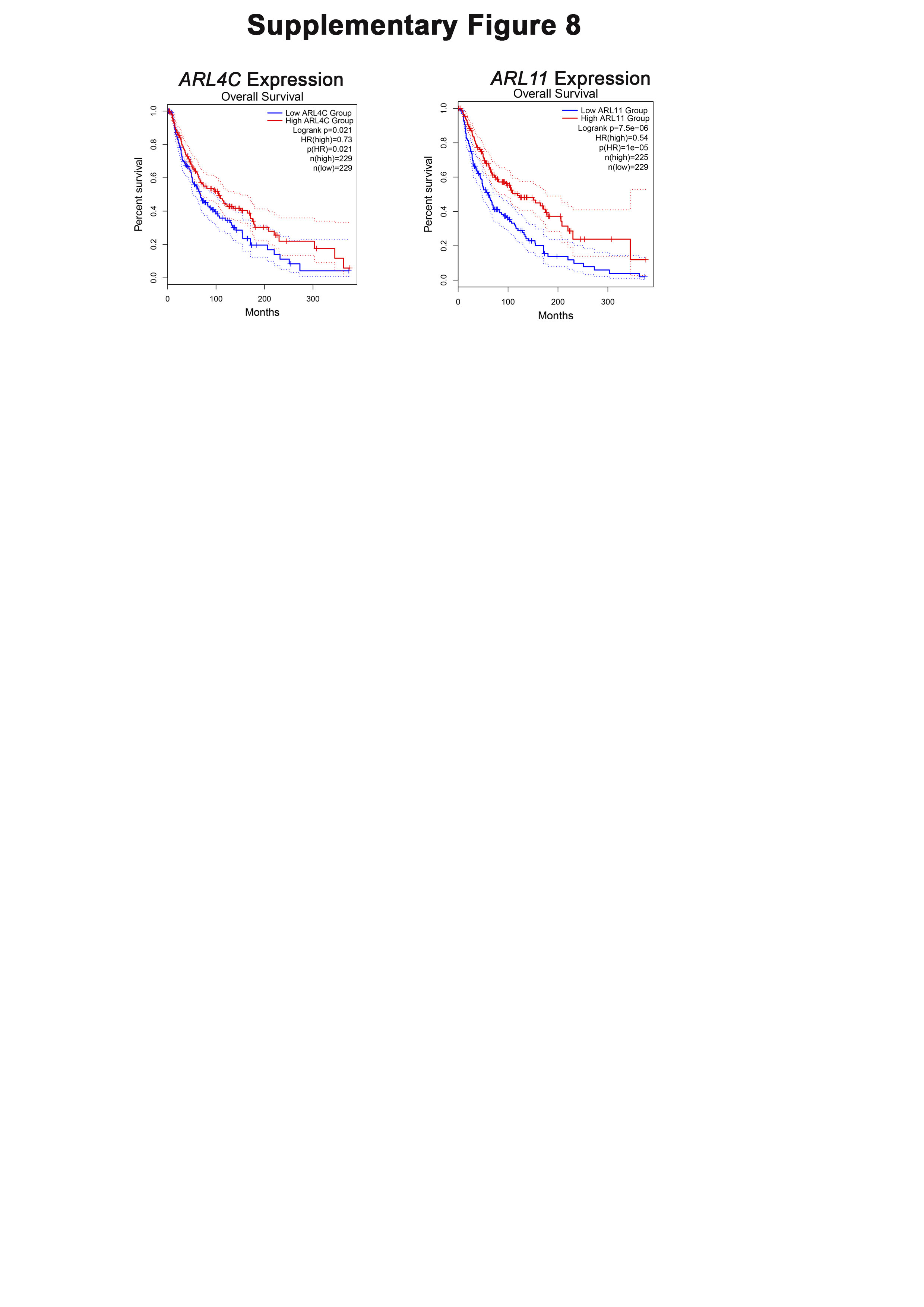

Supplement: Supplementary file 1 [file ijms-22-09260-s001.zip › Supplementary Figure S8.jpg]

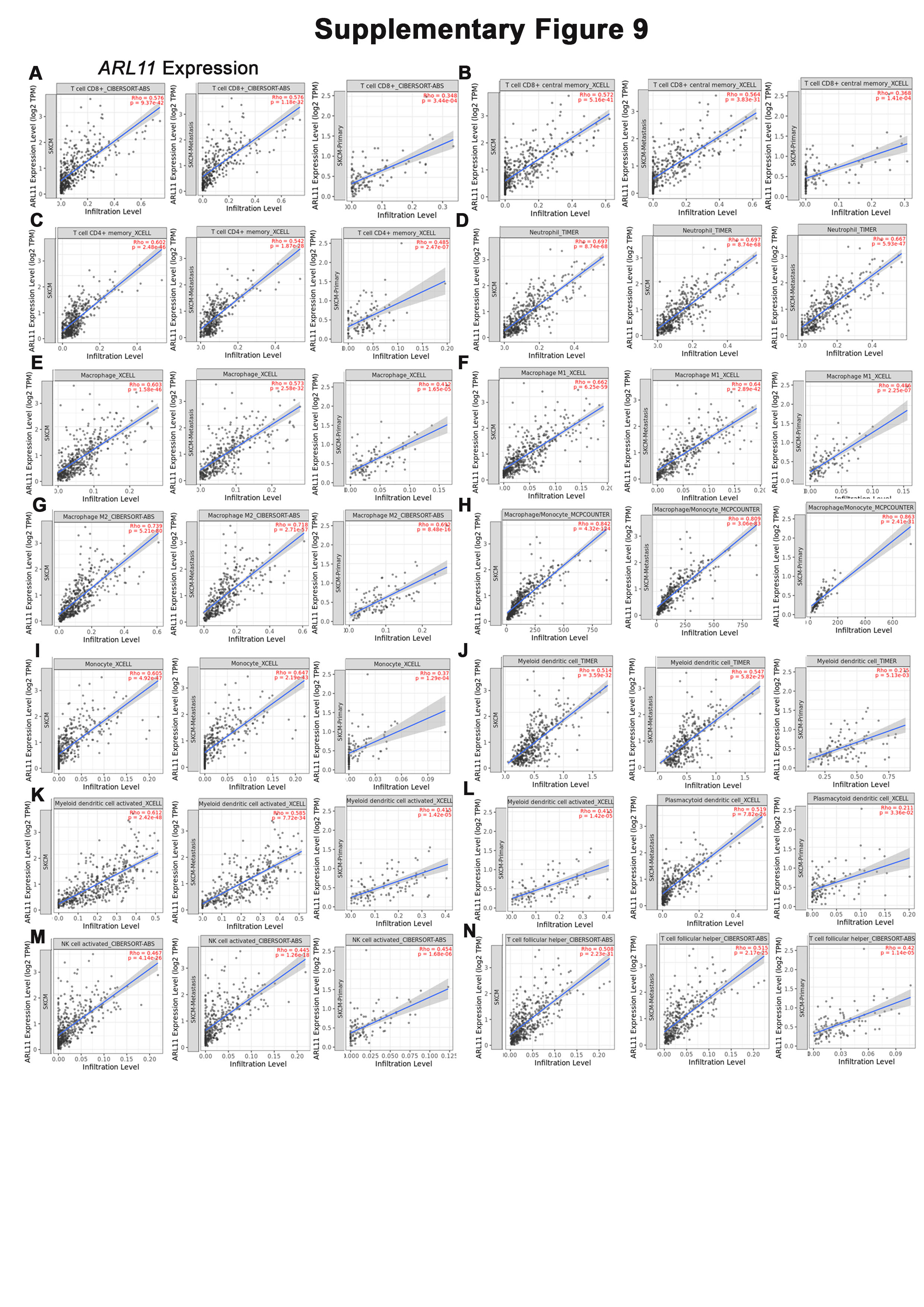

Supplement: Supplementary file 1 [file ijms-22-09260-s001.zip › Supplementary Figure S9.jpg]
